# Construction of a novel immune-related lncRNA signature and its potential to predict the immune status of patients with hepatocellular carcinoma

**DOI:** 10.1186/s12885-021-09059-x

**Published:** 2021-12-19

**Authors:** Min Deng, Jia-Bao Lin, Rong-Ce Zhao, Shao-Hua Li, Wen-Ping Lin, Jing-Wen Zou, Wei Wei, Rong-Ping Guo

**Affiliations:** 1 Department of Liver Surgery, Sun Yat-sen University Cancer Center, Guangzhou, China; 2 State Key Laboratory of Oncology in South China, Guangzhou, China; 3 Collaborative Innovation Center for Cancer Medicine, 651 Dongfeng East Road, Guangzhou, China; 4 Department of Health Management Center, Nanfang Hospital, Southern Medical University, Guangzhou, China

**Keywords:** Hepatocellular carcinoma, Immune-related lncRNA, Prognosis, Bioinformatics

## Abstract

**Background:**

The accuracy of existing biomarkers for predicting the prognosis of hepatocellular carcinoma (HCC) is not satisfactory. It is necessary to explore biomarkers that can accurately predict the prognosis of HCC.

**Methods:**

In this study, original transcriptome data were downloaded from The Cancer Genome Atlas (TCGA) database. Immune-related long noncoding ribonucleic acids (irlncRNAs) were identified by coexpression analysis, and differentially expressed irlncRNA (DEirlncRNA) pairs were distinguished by univariate analysis. In addition, the least absolute shrinkage and selection operator (LASSO) penalized regression was modified. Next, the cutoff point was determined based on the area under the curve (AUC) and Akaike information criterion (AIC) values of the 5-year receiver operating characteristic (ROC) curve to establish an optimal model for identifying high-risk and low-risk groups of HCC patients. The model was then reassessed in terms of clinicopathological features, survival rate, tumor-infiltrating immune cells, immunosuppressive markers, and chemotherapy efficacy.

**Results:**

A total of 1009 pairs of DEirlncRNAs were recognized in this study, 30 of these pairs were included in the Cox regression model for subsequent analysis. After regrouping according to the cutoff point, we could more effectively identify factors such as aggressive clinicopathological features, poor survival outcomes, specific immune cell infiltration status of tumors, high expression level of immunosuppressive biomarkers, and low sensitivity to chemotherapy drugs in HCC patients.

**Conclusions:**

The nonspecific expression level signature involved with irlncRNAs shows promising clinical value in predicting the prognosis of HCC patients.

**Supplementary Information:**

The online version contains supplementary material available at 10.1186/s12885-021-09059-x.

## Background

Hepatocellular carcinoma (HCC) is the fourth most common malignant tumor globally, and its incidence is increasing annually with poor survival [1, 2]. The primary relevant risk factors associated with the development of HCC include viral hepatitis, alcoholic liver disease, nonalcoholic fatty liver disease, aflatoxin exposure [3]. In recent years, chemotherapeutics have achieved encouraging results in treating HCC, especially immune checkpoint inhibitors (ICIs) [4]. With the recent success of clinical trials of immunotherapy, such as Checkmate 040, Keynote-224, and IMbrave150, ICIs such as nivolumab, pembrolizumab, and atezolizumab plus bevacizumab have been approved for the treatment of HCC [5–7].

Long noncoding RNAs (lncRNAs) are nonprotein-coding RNAs with a transcription length of more than 200 nucleotides [8]. Because lncRNAs are abundant, they often participate in human physiological processes and are closely related to the development of diseases [9–11]. In addition, lncRNAs have the ability to interact with molecules such as DNA, RNA, or protein to play enhancing or inhibitory roles [12]. Studies have reported that lncRNAs may participate in tumorigenesis through various molecular mechanisms [13, 14]. Recent work has demonstrated that lncRNAs can promote the malignant phenotypes of cancer by changing the genome or transcriptome level and varying the immune microenvironment [15]. LncRNAs can activate immune cells by expressing related genes, which leads to immune cells infiltrating tumors [16].

Immune cell infiltration markers in tumors show prospective predictive and prognostic value for tumor diagnosis, treatment, and survival evaluation [17–19]. Moreover, because lncRNAs have a close relationship with tumor immunity, the study of lncRNAs in combination with tumor immunity will help to establish these markers. The researches of Hong [20], Wei [21], and Qu [20] constructed models to predict the prognosis of HCC, pancreatic cancer, and clear cell renal cell carcinoma based on the immune-related lncRNAs (irlncRNAs) and risk scores, which have certain accuracy in predicting the prognosis of tumor patients. Our study built a novel signature constructed by irlncRNAs to predict HCC patients’ prognosis. IrlncRNAs, such as LINC01138, THUMPD3-AS1, AL365203, TBX2-AS1, have been confirmed to be related to the prognosis of HCC patients [21–24]. LINC01138 can promote cell proliferation, tumor invasion, metastasis and enhance protein stability by interacting with arginine methyltransferase 5 (PRMT5) [21]. TBX2 hypermethylation was associated with increased HCC risk [24]. While AC092535, FAM99A, AL355802, etc., have not been reported in HCC.

Generally, the accuracy of a tumor prediction model based on the combination of two biomarkers is better than that composed of a single gene [25]. To date, few models have been used to study the predictive role of lncRNAs and tumor immune-related cells in HCC [26, 27]. This study used a novel modeling algorithm, which does not require specific expression-level data, through pairing and iteration to establish an irlncRNA signature. Subsequently, we evaluated the diagnostic effect, predictive value, immune cell infiltration into tumors, and chemotherapy efficacy of this signature in HCC patients.

## Methods

### Data collection and expression analysis

The transcriptome data (RNA-seq) used for analysis in this study, corresponding to fragments per kilobase million (FPKM), was downloaded from the TCGA database (https://tcga-data.nci.nih.gov/tcga/). The GTF files used for subsequent analysis, distinguishing mRNA and lncRNA, were extracted from the genome database Ensembl (http://asia.ensembl.org). The ImmPort database (http://www.immport.org) was accessed to download a list of identified immune-related genes (ir-genes), serving a screening role to filter irlncRNAs through coexpression methods [28, 29]. The correlation between lncRNAs and ir-genes was analyzed. The inclusion criteria of the irlncRNAs were an immune gene correlation coefficient > 0.4 and a *p*-value < 0.001. The limma package of R was used to analyze the differential expression among irlncRNAs, and a log fold change (FC) > 1 and false discovery rate (FDR) < 0.05 were regarded as thresholds to distinguish different irlncRNAs (DEirlncRNAs).

### DEirlncRNA pairing

DEirlncRNAs were periodically single paired. When C was assumed to be equal to lncRNA A plus lncRNA B, a 0 or 1 matrix was constructed. When the lncRNA A expression level was higher than that of lncRNA B, C was defined as 1; otherwise, C was defined as 0. Then, the constructed matrix was further screened. When the expression level of lncRNA pairs was assumed to be 0 or 1, no relationship between pairing and prognosis was presumed because there was no specific level of pairing to correctly predict the survival outcome of patients [28, 29]. When the number of lncRNA pairs with an expression of 0 or 1 accounted for more than 30% of the total pairs, it was regarded as an effective match.

### Clinicopathological data acquisition

The clinicopathological information of HCC patients was collected from the TCGA database. After excluding cases with follow-up times less than 30 days and duplicate data, adequate data were extracted. Patients with tumor stage I (164 cases), stage II (77 cases), stage III (81 cases), and stage IV (3 cases) were recruited in this study.

### Establishing a risk model for evaluating the riskScore

The single-factor analysis was the first step in model generation. Then, LASSO regression with 10-fold cross-validation was carried out, and the *p*-value was 0.05. LASSO regression was performed for 1000 cycles, and 1000 random stimulations were set in each process. The next step was to record the frequency of each pair in the LASSO regression model, which was repeated 1000 times, and the pairs with a frequency greater than 100 times were selected for Cox proportional hazard regression analysis and model generation. The area under the curve (AUC) value was calculated, and the curve was plotted. When the maximum AUC value is that of the highest point of the curve, the calculation process is terminated, and the model is regarded as the best candidate [28, 29]. In this study, 1-year, 3-year, and 5-year receiver operating characteristic (ROC) curves of the risk model were drawn. The formula of the risk score for all clinical cases is as follows.

#### RiskScore = ${\varSigma}_{i=1}^k$ βiSi

The Akaike information criterion (AIC) value of each point of the 5-year ROC curve was used to determine the cutoff point to distinguish the high or low risk of risk scores.

### Risk model validation

Kaplan-Meier analysis was used to show the difference in survival rate between the high-risk group and the low-risk group. Then, the survival curve was plotted, and R was used to visualize the risk score of each case in the model. The R packages glmnet, survival, survivalROC, pbapply, surfminer, and pheatmap were utilized in these analyses. A chi-square test was conducted to investigate the relationship between the clinicopathological features and the model to validate the clinical application usefulness of the generated model [28, 29]. Then, the band chart was visualized and marked as follows: < 0.05 was marked *, < 0.01 was marked **, and < 0.001 was marked ***. Wilcoxon signed-rank test was performed to analyze the differences in riskScores between various clinicopathological feature groups. The results were demonstrated with box plots. Univariate and multivariate Cox regression analyses were of the risk score and clinicopathological features were performed to verify the possibility of an independent predictor of clinical prognosis for this model. The results are displayed in a forest plot. R packages, including ggupbr, survival, and pHeatmap, were used in these procedures.

### Study on tumor-infiltrating immune cells

The currently accepted methods for examining the immune cell infiltration status between samples from the LIHC of TCGA dataset were used to analyze the relationship between risk and immune cell features. These methods included XCELL, TIMER, QUANTISEQ, MCPCOUNTER, EPIC, CIBERSORT-ABS, and CIBERSORT. The infiltrating immune cell content analysis was followed by a comparison between the high-risk and low-risk groups was based on Wilcoxon signed-rank test. The results are shown in a box diagram. The relationship between infiltrating immune cells and riskScores was determined by Spearman correlation analysis. A lollipop chart was drawn to show the correlation coefficient of the results [28, 29]. *P* < 0.05 was considered to be a significant threshold. The Ggplot2 package in R was used to perform the procedure.

### The noteworthy relationship between the model and the clinical therapeutics

The IC50 of commonly used chemotherapy drugs in the LIHC dataset of the TCGA was calculated to assess the clinical application value of the model in the treatment of HCC. Chemotherapeutic medications such as bleomycin, doxorubicin, erlotinib, gemcitabine, methotrexate, mitomycin, paclitaxel, rapamycin, cisplatin, and sorafenib are commonly used in the treatment of various types of malignant tumors, as recommended by the AJCC recommendations [28, 29]. Wilcoxon signed-rank test was performed to assess IC50 between the two groups. The ggplot2 and pRRophetic packages in R were used to show the results.

### Expression analysis of immunosuppressive molecules related to ICIs

The relationship between the model and the gene expression level associated with ICIs was examined and visualized with a ggstatsplot and violin plot.

## Results

### Differential expression analysis of irlncRNAs

**Fig. 1:**
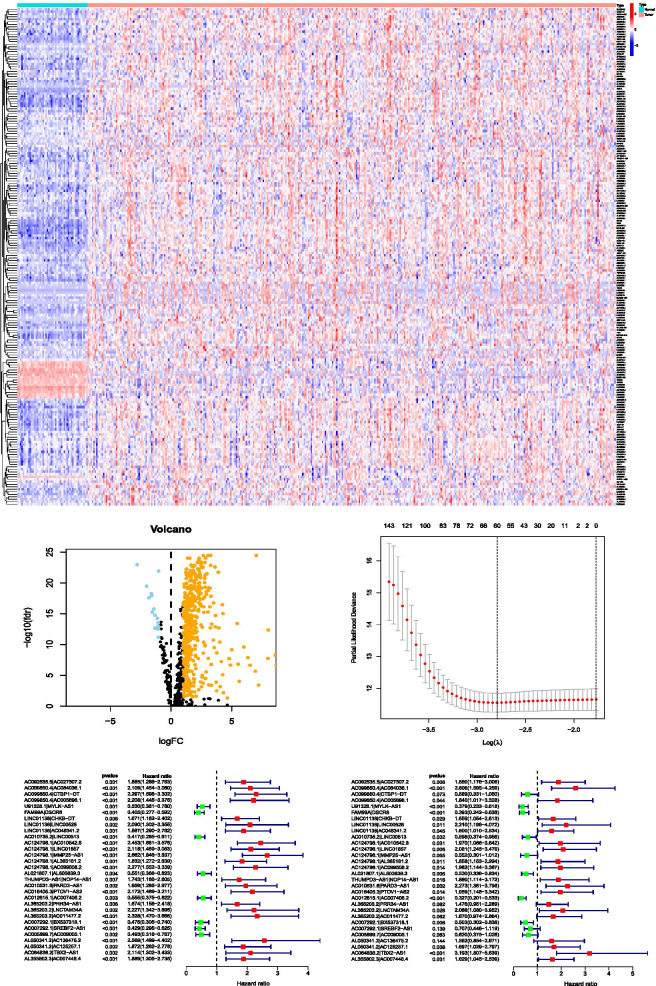
Establishment of a risk assessment model based on DEirlncRNA pairs. Differentially expressed immune-related lncRNAs (DEirlncRNAs). A heat map (**A**) and volcano plot (**B**) are displayed. Establishment of the LASSO regression (**C**). Thirty DEirlncRNA pairs are shown in a forest plot (**D** and **E**)

We downloaded transcriptome data of HCC from the TCGA database, including 50 nontumor tissues and 374 tumor tissues. The gene transfer format (GTF) files from Ensemble were used to annotate the accessed data. In total, 740 irlncRNAs were identified, of which 490 were classified as DEirlncRNAs. Among the DEirlncRNAs, 16 were downregulated, and 474 were upregulated (Fig. 1A, B, Tables S1, S2).

### DEirlncRNA pair screening and risk model construction

**Fig. 2:**
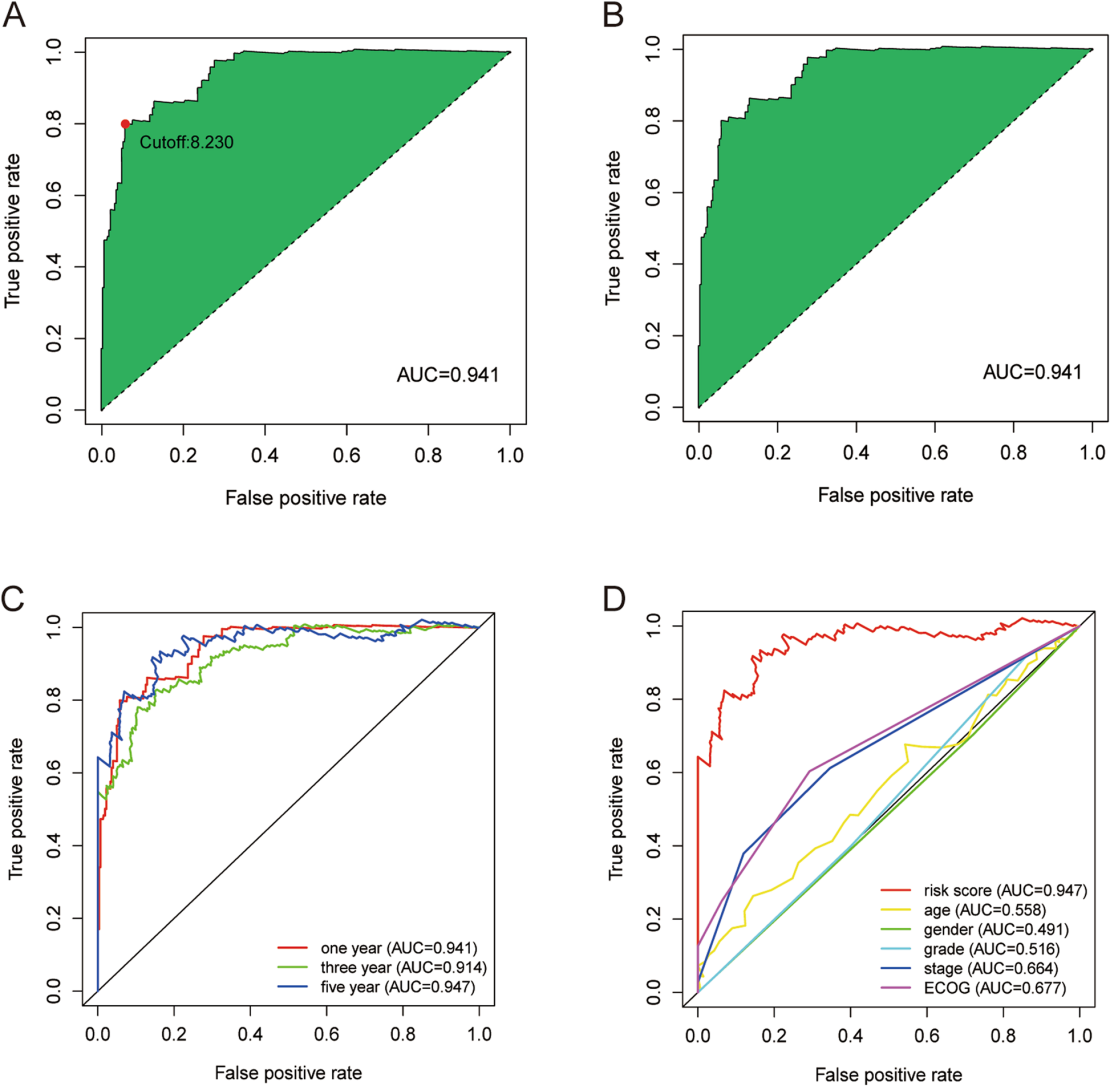
Establishment of a risk assessment model on the basis of DEirlncRNA pairs. The curve of each AUC value generated by the ROCs of 1009 DEirlncRNA pair models was drawn, and the highest point of AUC was determined. The maximum inflection point was the cutoff point acquired from the AIC. **A** and **B** The 1-year, 3-year, and 5-year ROC curves of the optimal model showed that all AUC values exceeded 0.91 (**C**). Compared with other common clinical features, the 5-year ROC curves showed a superior risk score (**D**)

A total of 10,344 valid DEirlncRNA pairs from the 490 DEirlncRNAs were identified by iteration loop and 0 or 1 matrix. A total of 1009 DEirlncRNA pairs were screened by a single factor test and modified LASSO analysis, of which 30 pairs were involved in the Cox model, as determined by the stepwise method. The results are shown in Fig. 1C, D, and E. Then, the AUC for each of the 1009 receiver operating characteristic (ROC) curves was calculated, and the curve was plotted. In addition, we found that the maximum AUC value was obtained when the highest point was equal to 0.941, and then the optimal DEirlncRNA pair was determined (Fig. 2A). Our study also used Akaike information criterion (AIC) values to determine the maximum inflection point as the cutoff point of the 5-year ROC curve (Fig. 2B). ROC curves at 1, 3, and 5 years were drawn to verify the optimality, which indicated that all AUC values exceeded 0.91 (Fig. 2C). Moreover, AUC values between the 5-year ROC curve and some common clinical parameters were also compared. (Fig. 2D). Furthermore, data of 343 patients with HCC were collected from the TCGA database, and the risk scores of all these patients were calculated. Then, the cutoff point was utilized to redifferentiate the high-risk group and low-risk group for verification.

### Application of the risk assessment model to a clinical evaluation

**Fig. 3:**
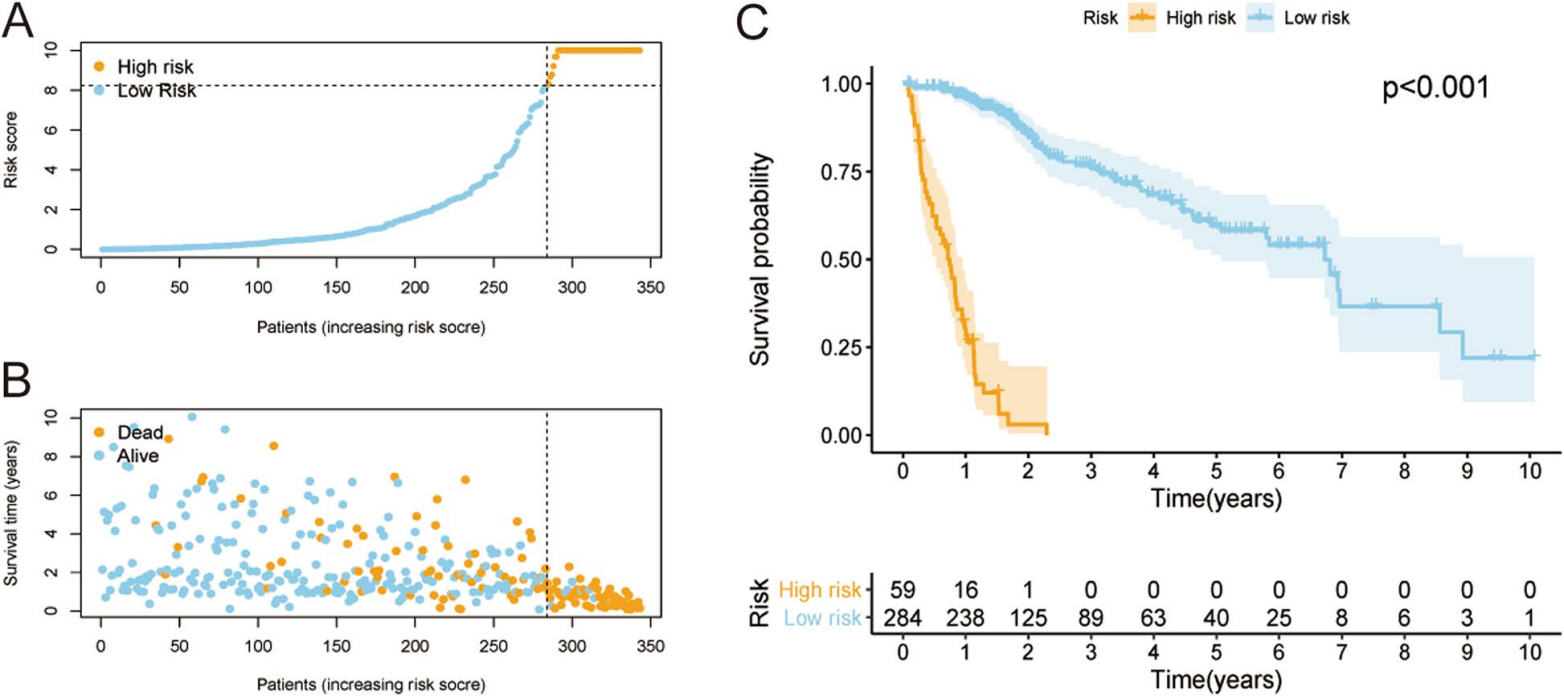
Prognostic power of the risk assessment model. Risk score (**A**) and survival outcome (**B**) of each case. Kaplan-Meier survival curve of the high-risk group and low-risk group (**C**)

**Fig. 4:**
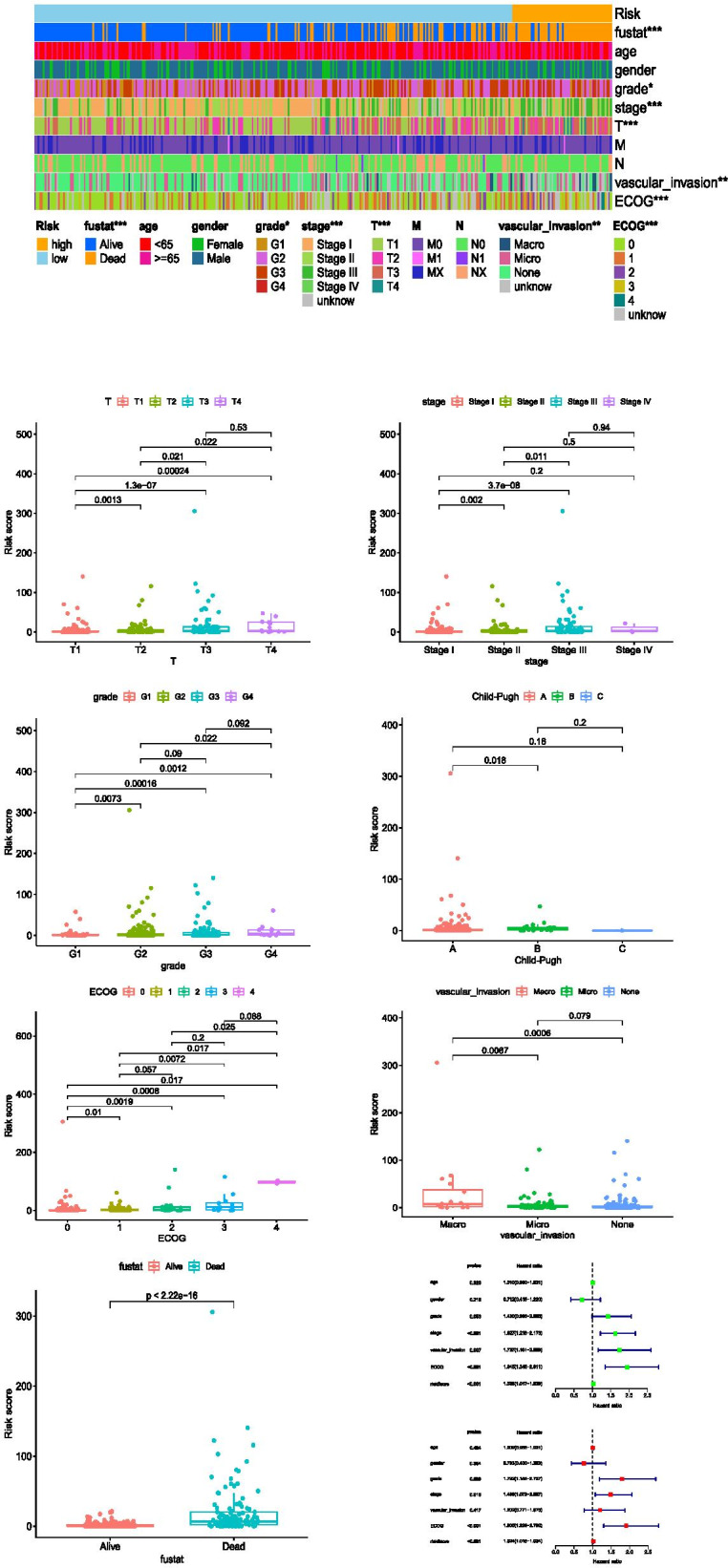
Application of the risk assessment model to a clinical evaluation. A strip diagram (**A**) and scatter plot show that T classification (**B**), tumor stage (**C**), tumor grade (**D**), Child-Pugh grade (**E**), ECOG (**F**), vascular invasion (**G**), and survival status (**H**) were significantly correlated with risk score. Univariate and multivariate Cox regression analyses were performed to analyze the clinicopathological features, and the results are shown in a forest map (**I**)

Fifty-nine cases and 284 cases were divided into the high-risk and low-risk groups according to the cutoff point. Figure 3A and B display the risk scores and survival rate of each case. These data indicate that patients’ clinical outcomes in the high-risk group were inferior to those in the low-risk group. The survival of the high-risk group was poorer than that of the low-risk group, as determined by Kaplan-Meier analysis (*p* < 0.001) (Fig. 3C). Next, a chi-square test was performed to explore the relationship between the risk of HCC and clinicopathological features. The stripping diagram (Fig. 4A) and the scatter plots examined through Wilcoxon signed-rank tests indicated that T classification, tumor stage, tumor grade, Child-Pugh grade, eastern cancer oncology group (ECOG) score, vascular invasion, and survival status (Fig. 4B-H) were significantly associated with risk. Subsequently, univariate Cox regression analysis indicated that there were significant differences in tumor stage (*p* < 0.001, HR = 1.627, 95% CI [1.218 – 2.173]), vascular invasion (*p* = 0.007, HR = 1.737, 95% CI [1.161 – 2.599]), ECOG (*p* < 0.001, HR = 1.945, 95% CI [1.346 – 2.811]), and risk score (*p* < 0.001, HR = 1.028, 95% CI [1.017 – 1.039]), while tumor stage (*p* = 0.018, HR = 1.488, 95% CI [1.072 – 2.067]), ECOG (*p* < 0.001, HR = 1.905, 95% CI [1.299 – 2.792]) and risk score (*p* < 0.001, HR = 1.024, 95% CI [1.015–1.034]) were determined to be independent prognostic predictors by multivariate Cox regression (Fig. 4I).

### Relationship between tumor-infiltrating immune cells, immune molecules and the risk model

**Fig. 5:**
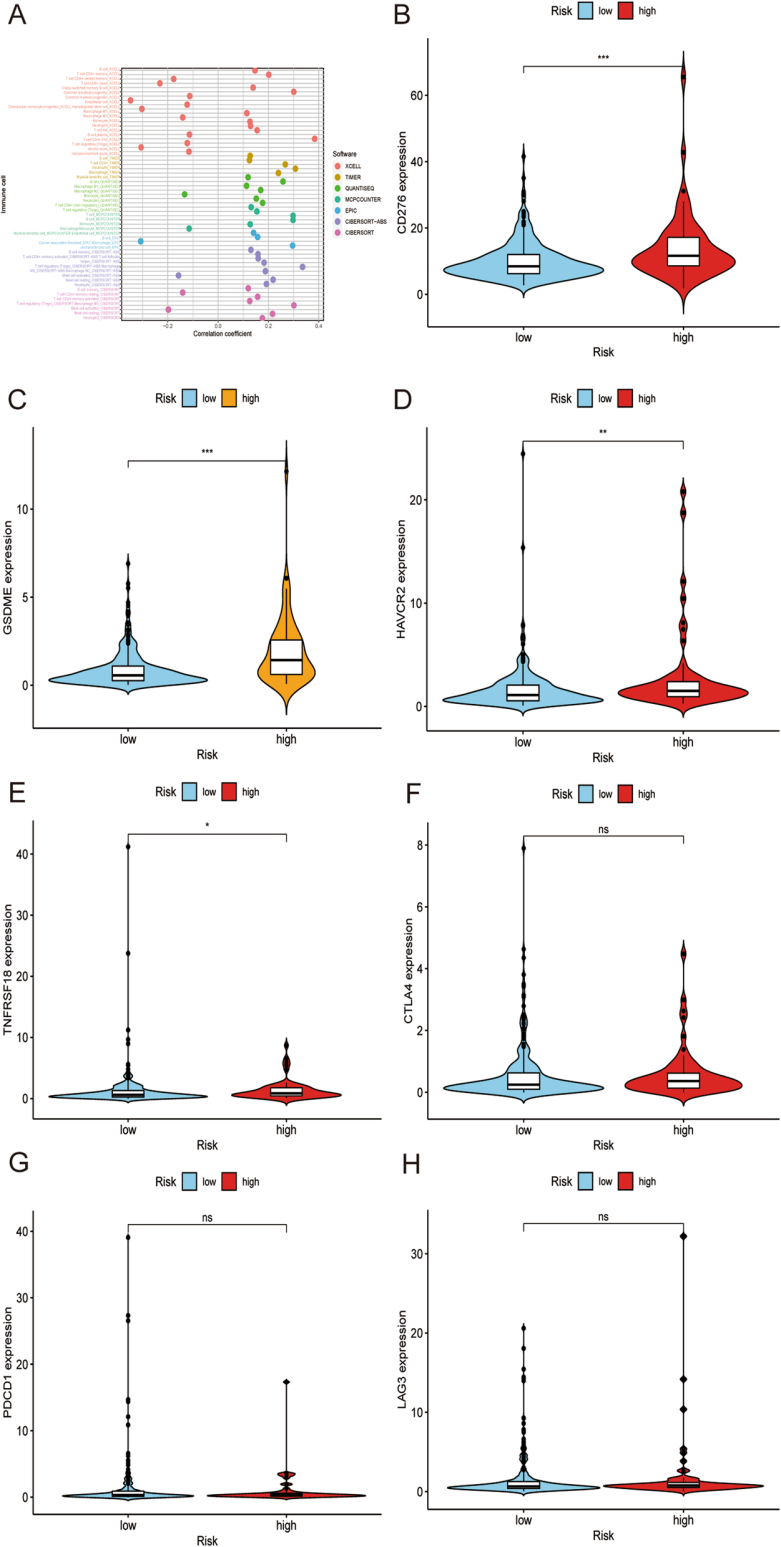
Estimation of tumor-infiltrating cells and immunosuppressive molecules with the risk assessment model. **A** Correlation between the high-risk group and tumor-infiltrating immune cells. High-risk scores were associated with the expression of CD276 (**B**), GSDME (**C**), HAVCR2 (**D**), and TNFRSF18 (**E**). There was no significant difference between the high-risk scores and the expression of immune-related genes, such as CTLA4 (**F**), PDCD1 (**G**), and LAG3 (**H**)

Since lncRNAs were initially associated with the ir-gene, we explored whether this model correlates with the tumor immune microenvironment. Our study found that there was a positive correlation between the high-risk group and tumor-infiltrating immune cells, such as B cells, neutrophils, and macrophages, but a negative correlation with CD8+ T cells, CD4+ T cells, and monocytes. Spearman correlation analysis was carried out in detail, and the results are shown in a lollipop diagram (Fig. 5A). Since ICIs currently play significant roles in the treatment of HCC, we explored the correlation between the risk model and ICI-related biomarkers. The results showed that high risk scores were associated with the expression of CD276 (*p* < 0.001), GSDME (*p* < 0.001), HAVCR2 (*p* < 0.01), and TNFRSF18 (*p* < 0.05) (Fig. 5B-E). There was no significant difference between the high-risk scores and ir-genes, such as CTLA4, PDCD1, and LAG3 (all *p* > 0.05, Fig. 5F-H).

### Correlation analysis of the risk model and chemotherapy drugs

**Fig. 6:**
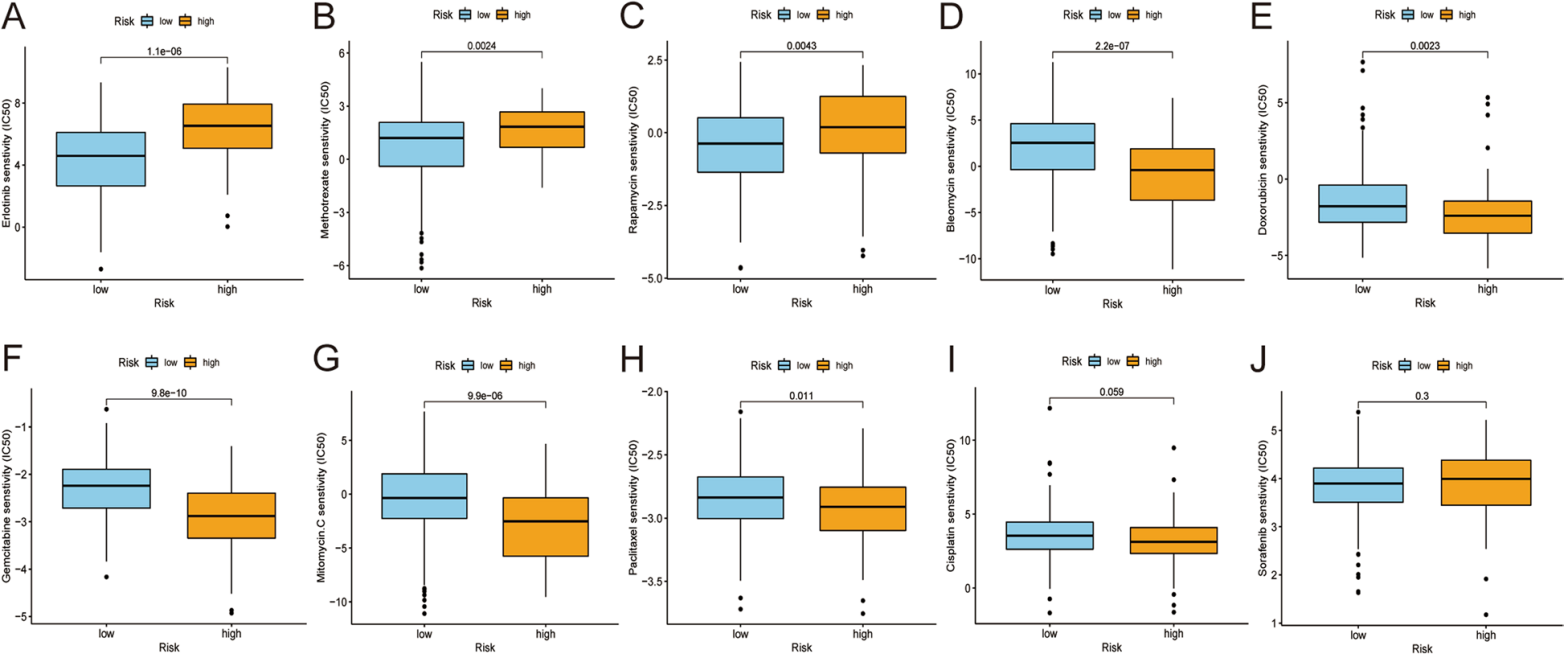
Relationship between risk scores and the IC50 of chemotherapeutics

In addition to checkpoint blocking therapy, we explored the association between the risk and efficacy of chemotherapy drugs commonly used for HCC. We found that the high-risk score was accompanied by a higher half inhibitory concentration (IC50) of chemotherapy drugs for erlotinib (*p* < 0.001), methotrexate (*p* = 0.0024), and rapamycin (*p* = 0.0043) and a lower IC50 for bleomycin (*p* < 0.001), doxorubicin (*p* = 0.0023), gemcitabine (*p* < 0.001), mitomycin (*p* < 0.001), and paclitaxel (*p* = 0.011), suggesting that this model can serve as a potential predictor for the sensitivity of chemotherapies (Fig. 6A-J).

## Discussion

It is necessary to improve the accuracy of prognostic markers for HCC patients. LncRNAs are closely related to normal physiological activities and the development of diseases [10, 30]. Furthermore, studies have demonstrated that lncRNAs play vital roles in tumor development and antitumor processes [31–33]. Recent studies have focused on investigating the potential relationship between coding genes and noncoding RNAs to predict patient prognosis with cancers [20, 34]. Unfortunately, the majority of these signatures were generated with the specific expression levels of transcripts. In our research, we ignored the specific expression levels of lncRNAs and utilized ir-gene pairing to generate a practical model with a combination of lncRNAs.

First, we downloaded the original information of lncRNAs from the TCGA database, and then a differential coexpression analysis was performed to catalog the DEirlncRNAs. The lncRNA pairs were verified by an improved cyclic single pair method along with 0 or 1 matrix. Second, univariate analysis and modified LASSO penalty regression were performed to determine DEirlncRNA pairs, procedures including cross-validation, multiple repetitions, and random stimulation. Then, we gained the optimum model by examining each AUC value of the ROC curve, and the optimum cutoff point was determined according to the AIC value of each point on the AUC to distinguish the high-risk and low-risk groups in the HCC dataset. Finally, the model was evaluated according to various parameters, such as survival rate, clinicopathological features, tumor-infiltrating immune cells, checkpoint-associated molecules, and chemotherapeutics.

The origin of lncRNAs may have the following four sources, mutation of a protein-coding gene, chromosomal rearrangement, duplications, and transposon insertion [35, 36]. Current research reveals that the phenotypic characteristics of lncRNAs regulation of cancer mainly include: cell proliferation, growth inhibition, cell migration, cell immortalization, angiogenesis, and cell viability [37]. The relationship between lncRNAs and tumors has received increasing attention [37–39]. Deng et al. established a model to predict HCC patient survival [40]. The method utilized in this study does require data on the specific expression level of each lncRNA; only pairs with high or low expression levels need to be detected. Therefore, the model is practical and straightforward in distinguishing high-risk or low-risk clinical cases. The lncRNAs included in this model are related to ir-genes; Therefore, these lncRNAs may regulate the immune microenvironment and the activation of immune cells.

Our research reveals that some of the DEirlncRNAs included in the modeling play vital roles in the malignant phenotype of many cancers, such as MYLK−AS1 [41, 42], THUMPD3 − AS1 [22], and DSCR8 [43], especially in the development of HCC. MYLK−AS1 promotes angiogenesis and HCC progression by targeting the miR-424-5p/E2F7 axis and activating the VEGFR-2 signaling pathway [42]. THUMPD3 − AS1 was associated with the cell cycle and can be used as a prognostic marker in hepatitis B virus-related HCC patients [22]. Wang et al. revealed that DSCR8 promotes the progression of HCC by activating the Wnt/b-catenin signaling pathway [44]. The established model can identify new biomarkers for further tumor-related studies.

To achieve better accuracy and effectiveness of risk prediction, this study used the improved method of the LASSO penalty model [45]. In addition, we determined the maximum value for an optimal model by calculating each AUC value and then compared it with other clinicopathological characteristics, further improving the modeling process. The AIC value was used to obtain the ideal cutoff point for model fitting; the median value was not used to discriminate risk. After using this new method to differentiate high-risk and low-risk groups, survival outcomes and univariate and multivariate analyses of clinicopathological features were reevaluated. Moreover, the sensitivity of chemotherapy drugs commonly used to treat HCC treatment was analyzed. The relationship between high-risk and low-risk groups and immune cell infiltration into tumors and the relationship between high-risk and low-risk groups and immune checkpoint-related genes were also studied, and the results indicated that this modeling algorithm has a good clinical application prospects.

The immune checkpoint blockade reaction is closely related to tumor-infiltrating immune cells [46]. Our research used seven commonly recognized methods to identify infiltrating immune cell to investigate the relationship between risk scores and tumor-infiltrating immune cells, including XCELL [47, 48], TIMER [49, 50], QUANTISEQ [51, 52], MCPCOUNTER [53], EPIC [54], CIBERSORT-ABS, and CIBERSORT [55, 56]. Due to the defects and complexity of these algorithms, they are rarely compared with each other. Through integration analysis, our findings show that DEirlncRNA pairs have a positive correlation with tumor-infiltrating immune cells such as B cells, neutrophils, and macrophages but are negatively correlated with CD8+ T cells, CD4+ T cells, and monocytes. Wang et al. demonstrated that the immune score can predict the efficacy of immunotherapy and chemotherapy [57]. IrlncRNA SATB2-AS1 can affect the tumor immune cell microenvironment and inhibit colorectal cancer metastasis [41]. LncRNA-EGFR can stimulate T regulatory cell differentiation and promote immune evasion in HCC [42]. Our model suggests that high risk is related to sensitivity to chemotherapy drugs such as methotrexate, rapamycin, bleomycin, doxorubicin, gemcitabine, mitomycin, and paclitaxel but not sensitivity to sorafenib. We believe that immunotherapy is more effective than traditional chemotherapy, mainly because immunotherapy can activate immune cell functions and promote tumor resistance by triggering active immunity. Tumor mutations can cause a large number of neoantigens to be released, which can be recognized by T cells and cause many immune cells to infiltrate into tumors [58–60].

We acknowledge that our study has some limitations. First, the research data were based on public databases. Some data were incomplete, such as some clinicopathological features and the sensitivity of drugs commonly used in the treatment of HCC; for instance, lenvatinib and oxaliplatin have not been analyzed. Second, the constructed model needs external verification because the expression level of each sample differs, which may lead to an unreliable final model. Third, this study did not analyze the expression level of these lncRNAs in immune cells. However, this research uses various methods to verify the new modeling algorithm and optimizes and analyzes it. Despite the lack of external data validation, from the analysis results, our model was acceptable. However, this study will be more convincing when external validation is performed. Therefore, our team will recollect clinicopathological data for subsequent studies and enlarge the sample size for further verification.

## Conclusions

Our research shows that an innovative signature established by irlncRNAs that does not require data on the expression levels of lncRNAs to predict HCC patient prognosis and may contribute to identification of patients who can benefit from antitumor immunotherapy.

## Supplementary Information

**Additional file 1.**

**Additional file 2.**

**Additional file 3.**

**Additional file 4.**

## Data Availability

Publicly available datasets were analyzed in this study, these can be found in The Cancer Genome Atlas (https://portal.gdc.cancer.gov/). Supplementary data associated with this article, named “Table S1”, “Table S2”, “Table S3”, and “Fig. S1A” have been uploaded.

## Abbreviations

HCC: Hepatocellular carcinoma
TCGA: The Cancer Genome Atlas
lncRNAs: Long non-coding RNAs
irlncRNA: Immune-related long non-coding ribonucleic acids
DEirlncRNA: Different expression irlncRNA
miRNA: microRNA
LASSO: Least absolute shrinkage and selection operator
AUC: Area under the ROC curve
AIC: Akaike information criterion
ROC: Receiver operating characteristic
ICIs: Immune checkpoint inhibitors
FPKM: Fragments perkilobase million
ir-genes: Immune-related genes
FC: Fold change
FDR: False discovery rate
GTF: Gene transfer format
ECOG: Eastern cancer oncology group
IC50: Half inhibitory concentration
