# Supplementary material for: Construction of a novel immune-related lncRNA signature and its potential to predict the immune status of patients with hepatocellular carcinoma
Source: BMC Cancer. 2021 Dec 19;21:1347. doi: 10.1186/s12885-021-09059-x (PMC8684648; doi:10.1186/s12885-021-09059-x)
Supplement: Supplementary file 1 — Additional file 1. [file 12885_2021_9059_MOESM1_ESM.pdf]

gene	conMean	treatMean	logFC	pValue	fdr
AC093673	3.163426	7.33399	1.21311	2.10E-11	4.58E-11
EPB41L4A-	1.832853	3.598996	0.973503	6.56E-11	1.39E-10
AC092535	1.036945	6.471465	2.641754	7.00E-19	3.81E-18
AC099850	0.183166	2.081554	3.506435	3.81E-18	1.81E-17
AL360219.	0.639637	0.537527	-0.25092	0.000104	0.000135
OSER1-DT	2.606592	4.802949	0.881756	1.71E-08	2.85E-08
U91328.1	0.472912	1.228562	1.377327	2.13E-14	6.34E-14
LINC0010€	0.37402	1.6431	2.135232	1.20E-15	4.04E-15
SMIM25	0.888395	0.63698	-0.47995	3.20E-09	5.67E-09
PPP1R14B	0.292719	1.214102	2.0523	1.39E-14	4.20E-14
AC100847	0.483925	0.636141	0.394563	0.490991	0.50393
SNHG6	11.70091	45.53758	1.960437	3.16E-21	3.25E-20
AC109322	0.245663	1.545494	2.653313	1.87E-27	3.47E-25
FAM99A	13.79846	5.52311	-1.32095	7.91E-14	2.14E-13
AL359921.	0.474474	1.734024	1.869724	1.10E-20	8.76E-20
AL031775.	1.3004	1.242449	-0.06577	0.133049	0.142277
AC007566	0.222305	0.549459	1.305468	4.23E-09	7.41E-09
DRAIC	1.705696	4.103461	1.266481	0.198899	0.209666
AL033397.	0.580005	1.620236	1.482067	0.487883	0.501436
AL109811.	0.891984	1.478697	0.729237	7.39E-08	1.17E-07
AC005840	0.281507	0.568707	1.014511	1.34E-08	2.27E-08
COA6-AS1	1.036828	3.322924	1.680276	7.34E-18	3.35E-17
AC007099	0.013203	0.612756	5.536428	1.02E-07	1.59E-07
LINC0033(	4.697271	8.12663	0.790835	2.75E-11	5.93E-11
AC025181	2.223789	2.91126	0.388624	0.022498	0.025149
LINC0113(	0.226353	1.055284	2.220986	5.96E-23	9.19E-22
TYMSOS	0.779702	2.927928	1.908885	3.63E-14	1.05E-13
AC005034	1.853131	3.231255	0.80213	4.73E-17	1.94E-16
AC010735	0.395278	1.056607	1.4185	1.02E-09	1.91E-09
AC084018	0.231808	1.102952	2.250367	9.36E-17	3.67E-16
UBR5-AS1	0.675682	1.83851	1.444121	1.57E-23	2.99E-22
AC090425	0.518182	0.789528	0.607532	0.003026	0.003526
AC124798	0.181986	0.61745	1.762497	0.025528	0.028322
CD27-AS1	1.341586	2.629844	0.971038	1.89E-17	7.92E-17
SH3BP5-A	0.322053	0.662067	1.039677	1.34E-09	2.47E-09
AL021807.	0.07637	0.629068	3.042135	1.32E-16	5.08E-16
THUMPD3	0.417553	1.434058	1.780072	6.85E-25	2.30E-23
AC010531	0.378412	0.970835	1.359269	1.94E-06	2.81E-06
AC016405	0.210645	0.929251	2.141253	3.03E-12	7.11E-12
AC023024	0.589243	0.844902	0.519923	0.372012	0.386099
LINC0229(	0.129878	0.703709	2.437816	8.98E-05	0.000117
AC012615	0.456284	1.737743	1.929209	1.10E-23	2.32E-22
HLA-DQB1	0.401763	0.681815	0.763037	0.037999	0.041906
AC009318	0.70692	0.959826	0.441227	0.000844	0.001012
AC023043	0.252395	0.890745	1.81933	6.65E-13	1.67E-12
AL109615.	0.045131	0.733589	4.022782	1.82E-11	4.01E-11
SNHG19	10.30831	26.1105	1.340823	1.57E-06	2.29E-06
AP001505.	1.278712	3.855314	1.592157	1.77E-12	4.27E-12
AL122010.	1.260369	1.588425	0.333751	0.045252	0.04961
AL365203.	0.682079	2.228084	1.707792	3.48E-15	1.12E-14
MAPKAPK	1.350742	3.967567	1.554503	8.83E-27	9.34E-25
AC067852	1.018286	1.759182	0.788762	8.56E-17	3.37E-16
AC007292	0.289337	1.002109	1.792216	4.50E-19	2.60E-18
TNFRSF14	0.383856	1.040593	1.438768	1.70E-06	2.47E-06
AC005899	0.189086	0.599536	1.664806	4.92E-10	9.45E-10
AL391422.	1.193266	2.507501	1.071334	1.53E-11	3.41E-11
ST20-AS1	0.629206	0.950838	0.595666	0.000432	0.00053

PSMB8-AS	2.205968	4.394326	0.99423	8.94E-08	1.40E-07
AL050341.	0.901121	2.179595	1.274267	7.24E-16	2.54E-15
HNF4A-AS	9.659838	5.926626	-0.70479	2.90E-09	5.17E-09
AC064836	0.488655	1.086648	1.152998	7.08E-10	1.34E-09
AL355802.	0.122382	0.583838	2.254182	5.07E-20	3.64E-19
LINC01018	21.23597	11.67817	-0.8627	1.11E-10	2.25E-10
ZFAS1	6.45919	19.53775	1.596839	1.38E-17	5.96E-17
MUC12-AS	0.061443	0.620857	3.336938	0.000339	0.000422
AC091729	1.120439	2.851027	1.347418	9.19E-18	4.10E-17
FLVCR1-D	0.231485	1.111709	2.263791	2.61E-19	1.61E-18
LINC00909	0.794398	1.456658	0.874728	1.20E-12	2.96E-12
POLR2J4	0.618527	1.265251	1.032515	1.01E-16	3.92E-16
AL391834.	0.960269	1.609308	0.744931	2.57E-08	4.22E-08
SLC25A25-	0.693352	1.842662	1.410132	8.60E-10	1.62E-09
AC073611	0.204749	0.733007	1.839972	1.15E-18	5.94E-18
ASB16-AS	0.835192	2.624523	1.651876	2.86E-25	1.25E-23
AL031673.	0.495194	1.843383	1.896291	2.06E-19	1.30E-18
AC008764	0.420982	1.184437	1.492371	8.15E-11	1.68E-10
BX322562.	0.793129	2.019644	1.348473	0.016542	0.018631
LINC01128	0.779075	0.756961	-0.04154	0.03052	0.033709
AL358472.	0.389859	0.892932	1.195597	4.37E-16	1.61E-15
LINC01278	1.190393	2.270893	0.931821	3.53E-16	1.31E-15
AC008736	0.374566	1.090306	1.541439	2.29E-12	5.48E-12
TRIM52-A	2.198441	4.953459	1.171956	1.09E-15	3.71E-15
AL391832.	0.853963	1.693342	0.987628	0.000102	0.000132
AC010336	1.033911	0.689401	-0.5847	6.00E-08	9.63E-08
AC016394	0.13097	0.719759	2.45828	1.09E-25	5.35E-24
AC005225	0.992931	0.916243	-0.11596	0.00281	0.00328
LINC01503	0.556853	1.431528	1.362188	0.002721	0.003186
AC009779	1.793328	3.783868	1.077222	1.42E-16	5.45E-16
PXN-AS1	0.720148	1.894678	1.395587	4.65E-21	4.59E-20
PRR34-AS	0.935418	3.428075	1.873715	2.02E-13	5.28E-13
PVT1	0.118649	0.929567	2.969859	2.90E-20	2.15E-19
AC004832	1.433334	1.143837	-0.32549	2.16E-05	2.95E-05
SNHG25	0.651616	5.018619	2.945195	1.99E-23	3.60E-22
AC004687	0.196521	0.638455	1.699901	1.53E-06	2.25E-06
LINC02453	1.131477	1.01446	-0.1575	0.000108	0.00014
ZFPM2-AS	0.053865	1.493454	4.793167	1.20E-08	2.05E-08
AC104825	0.409677	0.974514	1.250195	1.07E-10	2.19E-10
AC009407	3.991088	10.44651	1.388166	3.63E-09	6.39E-09
AC103691	0.443405	0.929335	1.067575	8.70E-08	1.37E-07
AL390198.	0.425944	1.27319	1.579712	0.000142	0.000181
PITPNA-A	1.65081	4.433619	1.425311	6.23E-16	2.23E-15
LINC00294	0.862224	1.874373	1.120273	2.84E-18	1.38E-17
NAPA-AS1	0.401076	0.635507	0.664031	0.000407	0.000501
AL162582.	0.133943	1.827624	3.770279	1.23E-09	2.28E-09
AC010280	3.545021	0.881215	-2.00823	3.36E-21	3.41E-20
AC009159	1.784355	1.131107	-0.65767	3.47E-10	6.76E-10
AC092687	0.468614	0.861802	0.878957	0.023648	0.026354
AL160006.	0.215474	0.817242	1.923247	7.63E-22	8.96E-21
AC010969	0.527831	1.193937	1.177578	1.85E-17	7.84E-17
AL354920.	1.737402	2.197274	0.338783	0.014108	0.016012
AC027117	10.92721	8.288939	-0.39867	6.10E-05	8.02E-05
AC010542	0.23622	0.873335	1.886403	1.17E-18	6.03E-18
AL021328.	1.38585	0.473786	-1.54846	1.47E-16	5.60E-16
AC007383	0.962149	1.559947	0.697165	3.60E-06	5.08E-06
A2M-AS1	0.601273	0.736931	0.29351	0.172004	0.181833
AC009283	1.814618	4.569689	1.332431	7.56E-19	4.08E-18

AC024896	3.38072	4.608285	0.446899	0.072907	0.078992
AC244090	3.362677	9.028119	1.424815	1.98E-17	8.21E-17
RNU6ATA(	0.391697	0.60752	0.633191	0.249572	0.261591
C8orf49	0.470504	0.665351	0.499906	0.32048	0.334022
AC008267	2.82838	6.126457	1.115077	6.67E-11	1.40E-10
AC147067	0.332911	0.811313	1.285123	1.07E-05	1.48E-05
AC009237	1.338443	3.017227	1.172668	1.64E-15	5.38E-15
AC073508	1.37224	2.302489	0.746662	7.54E-13	1.88E-12
AC002467	0.748915	1.413707	0.91661	2.25E-08	3.72E-08
LINC0177(	0.756309	1.989617	1.395443	2.32E-07	3.52E-07
AC002456	0.52978	1.384336	1.385728	8.84E-05	0.000115
AC136475	0.532915	1.350711	1.341741	7.27E-13	1.82E-12
AC138696	0.58664	2.541744	2.11527	2.28E-20	1.72E-19
AL391095.	15.52089	9.634086	-0.68799	6.10E-08	9.75E-08
AC008443	0.643931	1.837001	1.512374	4.13E-10	7.98E-10
DBH-AS1	5.001625	7.372181	0.559694	0.412095	0.426504
LINC0127:	0.53391	1.066683	0.998462	2.74E-07	4.14E-07
SERTAD4-	0.052423	0.667172	3.66979	5.39E-16	1.94E-15
AC083841	3.929186	7.690374	0.968823	0.030425	0.033654
AC132872	0.248568	0.632864	1.348257	1.93E-09	3.48E-09
AC084036	1.128049	3.565437	1.660249	4.51E-16	1.65E-15
CTBP1-DT	0.738001	1.797603	1.28438	1.00E-21	1.16E-20
AL162413.	0.01103	4.245629	8.588431	3.05E-09	5.42E-09
AL121944.	1.261878	2.527907	1.002371	6.35E-11	1.35E-10
AC099343	0.733103	1.106968	0.594526	0.011545	0.013163
AC007448	0.264069	0.772098	1.547867	1.03E-09	1.94E-09
AL450384.	1.39597	2.596007	0.895026	0.000352	0.000437
BX537318.	0.477849	1.423758	1.575077	6.92E-21	6.17E-20
ARRDC1-/	1.527505	3.569038	1.224358	8.45E-24	1.84E-22
LINC0142(	0.074323	0.835773	3.491224	8.19E-12	1.87E-11
AC007406	0.725661	1.128959	0.637625	7.14E-06	9.96E-06
AL445524.	1.830088	12.66058	2.790358	8.48E-26	4.82E-24
AC009113	0.598184	0.968462	0.695107	0.000681	0.000824
AC092368	1.373768	2.229316	0.698463	1.32E-08	2.23E-08
DLG5-AS1	0.179582	1.385736	2.947938	1.56E-23	2.99E-22
DLGAP1-/	1.271828	3.068002	1.270396	2.09E-10	4.13E-10
KIAA1671-	2.059971	1.690366	-0.28529	0.00024	0.000305
EGILA	0.37163	0.561984	0.596664	0.062313	0.067711
AL049840.	2.057305	5.375175	1.385556	1.63E-19	1.07E-18
CYTOR	0.722748	4.130732	2.514832	8.43E-21	7.16E-20
AC011445	0.303189	2.805598	3.210017	8.52E-21	7.16E-20
AC110285	0.228376	1.539482	2.752965	3.56E-19	2.16E-18
AC083880	0.202429	0.676264	1.740175	1.55E-14	4.67E-14
MINCR	0.769633	3.339484	2.117382	5.23E-21	4.84E-20
AL023803.	0.581269	1.525726	1.392218	2.43E-05	3.28E-05
AL928654.	0.307821	0.91663	1.574249	9.11E-12	2.07E-11
LINC0131!	0.163606	0.665645	2.024525	4.72E-13	1.20E-12
AP000424.	0.121326	0.591554	2.285618	0.001875	0.002199
OIP5-AS1	5.017977	6.431934	0.358147	0.000914	0.001094
AC022150	0.203157	0.547938	1.431416	0.000317	0.000395
AP001160.	0.788779	1.188294	0.591199	3.92E-07	5.90E-07
AL513165.	2.303908	4.042728	0.811246	5.54E-07	8.30E-07
CHKB-DT	0.521489	1.286361	1.302586	2.63E-16	9.88E-16
AC006504	0.201836	0.729382	1.853491	7.10E-16	2.51E-15
AC006213	0.253245	0.839264	1.728594	3.29E-10	6.43E-10
PRKAR1B-	0.312737	0.842778	1.430202	1.88E-08	3.14E-08
AC127024	0.187299	0.878004	2.228885	9.47E-21	7.62E-20
ADORA2A	3.827802	6.33442	0.726696	0.000148	0.000189

AC026471	0.700185	1.612986	1.203926	3.73E-14	1.07E-13
MIR210HG	0.439851	1.419384	1.69018	0.000416	0.000512
AL450998.	0.68925	1.244928	0.852962	4.11E-08	6.68E-08
EBLN3P	3.946758	5.994877	0.603062	3.52E-06	4.99E-06
AC009065	0.518518	1.219769	1.234141	5.97E-15	1.87E-14
AL359643.	0.336933	0.836039	1.311108	3.18E-14	9.23E-14
AL139260.	0.382613	0.761035	0.992078	5.61E-05	7.40E-05
AL136295.	0.650119	1.238842	0.930218	7.95E-13	1.97E-12
AC083799	3.660389	5.023905	0.456812	0.000392	0.000484
AC136475	0.440161	1.324741	1.589607	0.011424	0.013046
AL118516.	1.739653	3.558272	1.032377	2.12E-10	4.19E-10
DHRS4-AS	7.080514	5.831733	-0.27993	0.001202	0.001432
AC006942	0.383483	0.698022	0.864109	0.000592	0.00072
AC120042	0.648836	0.615657	-0.07573	0.000112	0.000145
AL158071.	0.203916	0.619443	1.602999	3.73E-10	7.22E-10
AL022328.	0.317449	0.790558	1.316347	1.97E-11	4.32E-11
AFAP1-AS	0.005129	1.323426	8.011325	1.66E-13	4.40E-13
SNHG11	1.670991	5.161074	1.626967	3.39E-23	5.70E-22
LINC0020	0.289346	1.405556	2.280276	1.03E-21	1.17E-20
AC010331	0.119088	0.606362	2.348153	7.63E-22	8.96E-21
NCK1-DT	0.636228	1.416334	1.154545	2.25E-19	1.41E-18
AL021707.	0.616379	0.933644	0.599054	0.013354	0.01518
AC011477	0.378295	0.986702	1.383104	2.25E-07	3.42E-07
AL354892.	1.056558	2.284709	1.112638	8.24E-08	1.30E-07
AC091271	0.670871	1.479532	1.141033	2.30E-08	3.79E-08
ELOA-AS1	0.540842	0.919986	0.766406	6.86E-06	9.58E-06
AC012467	0.571004	1.269549	1.152742	5.50E-20	3.91E-19
AP000240.	0.213293	0.639438	1.583968	1.17E-09	2.17E-09
Z95115.1	0.339388	1.009343	1.572409	5.82E-14	1.61E-13
SNHG30	1.354942	3.944495	1.541609	8.44E-19	4.46E-18
AP001816.	2.416077	3.83216	0.665491	2.85E-06	4.08E-06
DM1-AS	0.817458	1.348155	0.72177	3.14E-06	4.46E-06
GIHCG	0.38911	1.925016	2.306622	3.99E-24	9.83E-23
TBX2-AS1	0.171174	0.777199	2.182823	9.24E-12	2.08E-11
AL590617.	1.281761	1.779648	0.473464	0.843656	0.852876
AC104958	3.574543	12.10274	1.759503	3.24E-14	9.37E-14
AC103706	0.254451	1.342626	2.399596	7.37E-21	6.42E-20
AL132989.	0.295301	0.66163	1.163838	1.61E-07	2.47E-07
AC005586	0.291505	0.867054	1.572601	8.31E-13	2.06E-12
AP003068.	0.344697	0.649664	0.914365	1.74E-06	2.53E-06
AC074032	0.567577	0.676058	0.252331	0.891023	0.897085
ST7-AS1	0.206386	0.545946	1.403417	9.95E-11	2.05E-10
AC012409	1.705277	0.83098	-1.03712	2.68E-12	6.37E-12
AC104113	0.486284	1.070232	1.138052	9.24E-12	2.08E-11
AC015982	0.464077	0.946198	1.027776	1.55E-10	3.11E-10
AC009506	0.642823	0.926796	0.527831	0.00026	0.000328
RPARP-AS	0.721191	1.489844	1.046709	4.39E-14	1.25E-13
CCDC18-A	0.248727	1.024184	2.041839	6.26E-23	9.45E-22
AC007114	1.295318	2.730659	1.075942	6.19E-11	1.32E-10
PIK3CD-A	0.216875	0.722154	1.73544	0.227761	0.239068
AC015912	0.368842	1.482856	2.007305	7.30E-11	1.52E-10
AC055720	6.403666	9.265742	0.533008	0.058529	0.063788
CAMTA1-I	0.242566	0.593918	1.291887	1.16E-07	1.81E-07
LINC0276	1.389425	1.195981	-0.21629	9.30E-06	1.29E-05
AC108488	0.456415	0.871203	0.932663	6.55E-10	1.25E-09
AC027601	0.371466	0.646598	0.79964	0.164379	0.17452
AC026369	0.198162	0.548709	1.469362	1.89E-13	4.97E-13
CNNM3-D	0.637204	1.082656	0.764747	4.74E-05	6.28E-05

TAT-AS1	0.427066	0.881869	1.046106	0.009454	0.010829
EMSLR	1.301788	2.217777	0.768619	0.193372	0.20413
AC092490	0.053888	0.711839	3.723513	9.73E-05	0.000127
AP002748.	0.545956	1.294132	1.245128	2.48E-14	7.29E-14
SSTR5-AS:	0.009849	1.461736	7.213482	0.000309	0.000387
ITGB1-DT	0.566653	1.563889	1.464602	0.00106	0.001267
AL391121.	0.625961	1.014516	0.696648	0.000719	0.000867
MAP3K2-[	0.419095	1.008296	1.266572	1.30E-09	2.40E-09
AL354733.	0.26252	0.553279	1.07558	0.001074	0.001282
ANKRD10-	0.853368	2.269042	1.410844	1.66E-07	2.54E-07
AC005696	0.36597	0.830937	1.183012	1.47E-08	2.46E-08
AC008610	0.197255	1.030347	2.384993	9.02E-19	4.70E-18
AC008669	0.444452	0.697988	0.651175	0.005153	0.005958
AC112491	0.980106	1.973378	1.009658	0.001643	0.001939
AC008549	23.38095	11.31306	-1.04734	3.88E-14	1.11E-13
AC124016	0.256978	0.622836	1.27721	6.96E-07	1.04E-06
AC074117	0.472669	1.294549	1.453547	4.76E-21	4.64E-20
AC008771	1.045184	2.529414	1.275047	5.02E-19	2.88E-18
NOP14-AS	0.508031	1.086813	1.097115	4.54E-21	4.54E-20
GAS5	6.027653	33.72981	2.484356	3.75E-25	1.39E-23
TOB1-AS1	0.497459	0.924336	0.893838	1.26E-07	1.95E-07
AC093297	1.663034	2.969431	0.836369	5.65E-14	1.58E-13
LINC0170:	0.124767	1.046643	3.068464	2.05E-20	1.57E-19
LINC0052ε	0.127875	0.637619	2.317958	2.50E-20	1.87E-19
DANCR	4.42438	11.78659	1.413599	4.06E-09	7.13E-09
AL355102.	0.350582	2.513658	2.841965	2.42E-05	3.28E-05
AL117336.	0.237875	0.826471	1.796759	5.49E-19	3.08E-18
ZNF529-A	0.294109	0.914214	1.63618	1.19E-20	9.40E-20
AC008966	0.529005	1.045978	0.983499	3.71E-08	6.04E-08
LINC01857	0.259608	0.534095	1.040763	0.000205	0.000261
VPS13B-D	0.732605	2.321344	1.663853	4.04E-15	1.29E-14
AC145207	0.195689	0.684148	1.805747	4.30E-19	2.53E-18
SNHG18	1.080307	1.030957	-0.06746	0.000291	0.000366
LINC0102:	2.022843	3.454913	0.772265	7.86E-05	0.000103
AL035071.	0.803633	1.998843	1.314556	6.45E-15	2.01E-14
AC048341	0.215025	1.087921	2.338998	3.03E-21	3.20E-20
AC026740	0.138208	1.35502	3.293399	2.29E-19	1.42E-18
AL451165.	2.105299	4.605082	1.129202	6.73E-12	1.54E-11
AL121992.	3.012165	2.699098	-0.15832	0.005153	0.005958
LINC0100	0.201823	0.842962	2.062378	2.09E-22	2.76E-21
AC084824	0.42867	1.024414	1.25686	1.66E-13	4.40E-13
AC020558	0.599658	0.755113	0.332553	0.477176	0.491113
AC027644	1.313399	2.823769	1.104317	8.82E-08	1.39E-07
AC068338	1.653491	1.524388	-0.11728	0.001874	0.002199
LINC0109:	30.82677	4.466404	-2.787	2.29E-25	1.06E-23
AC012146	0.325398	1.504259	2.208774	3.99E-20	2.90E-19
AC005076	0.334897	0.643741	0.942764	2.82E-06	4.04E-06
AL162274.	0.701714	0.883491	0.332332	0.200623	0.211182
THAP9-AS	1.36025	2.527675	0.893939	7.67E-11	1.59E-10
TIPARP-A!	0.774585	1.421901	0.876326	2.94E-06	4.19E-06
AC093110	0.465059	0.531747	0.193327	0.77647	0.788186
AL450326.	0.961751	1.067818	0.15093	0.848466	0.856569
AL161669.	0.361577	1.433957	1.987625	5.16E-15	1.62E-14
PANK2-AS	0.23517	0.654008	1.475604	4.43E-13	1.14E-12
AC015922	0.742125	0.546957	-0.44023	4.18E-08	6.77E-08
AC118553	0.691819	1.059149	0.614439	0.005926	0.00683
LINC0203!	0.291287	0.618881	1.087221	1.71E-10	3.40E-10
CIRBP-AS:	0.658937	0.925411	0.489954	2.22E-07	3.38E-07

SNHG15	0.907477	1.884032	1.05389	8.83E-06	1.23E-05
AC006042	0.156822	0.648316	2.047564	4.47E-09	7.80E-09
AC060780	0.228525	0.809819	1.82525	2.87E-12	6.79E-12
PARD3-AS	0.21016	0.590742	1.491039	1.97E-08	3.27E-08
AC011450	0.492141	0.903762	0.876871	2.30E-05	3.13E-05
GASAL1	0.391538	0.598277	0.611664	0.013127	0.014945
AC010547	2.315825	1.131974	-1.03269	5.12E-24	1.18E-22
C2-AS1	0.245822	0.628038	1.353239	6.06E-08	9.70E-08
AC132938	2.115054	2.516497	0.250723	0.656006	0.669579
AC083843	0.297522	0.754353	1.342241	1.38E-08	2.31E-08
AC120053	0.55038	1.628419	1.564972	6.09E-20	4.29E-19
SNHG14	0.211369	0.637834	1.593418	0.006129	0.007054
AC004540	1.749296	0.567111	-1.62507	8.08E-20	5.53E-19
AL121899.	0.318121	2.390599	2.909724	1.11E-19	7.40E-19
AC098487	0.550355	0.954852	0.794914	0.000311	0.000389
LINC0216	0.00121	1.021286	9.721556	1.64E-14	4.91E-14
AC090150	1.176018	2.958685	1.331046	0.883244	0.890464
AC245297	0.75939	1.489782	0.972188	5.30E-07	7.95E-07
AC104083	1.807958	1.773652	-0.02764	0.000546	0.000665
AC093278	0.463231	0.908582	0.971884	3.42E-10	6.68E-10
MYLK-AS1	0.097409	0.699674	2.844562	7.53E-27	9.34E-25
AC115618	2.291425	4.702419	1.037158	5.94E-11	1.27E-10
AP003352.	0.373765	1.541082	2.043742	2.65E-23	4.56E-22
DICER1-A	0.353131	0.754735	1.095769	1.27E-11	2.85E-11
AC010326	1.238888	2.959211	1.256167	4.83E-17	1.96E-16
SNHG29	18.16456	37.66017	1.051913	5.89E-08	9.48E-08
AL391056.	0.022611	0.671633	4.892558	1.40E-07	2.16E-07
LINC0187	1.221096	1.772902	0.537936	0.304005	0.317746
RBPMS-A	0.403163	0.737624	0.871524	0.000137	0.000176
LINC0157	2.804924	4.617331	0.719098	6.35E-11	1.35E-10
AC244100	0.667821	0.646731	-0.0463	0.019745	0.022205
SREBF2-A	0.190006	0.929223	2.289977	2.58E-26	2.12E-24
HSD11B1-	1.33292	3.547031	1.412022	0.000442	0.000541
AC009022	0.334902	0.709271	1.082599	1.28E-10	2.59E-10
HCG18	0.341767	0.924909	1.436299	2.01E-18	1.01E-17
AC005332	1.737848	4.635553	1.41544	2.31E-18	1.14E-17
SEMA3B-A	0.387422	1.398511	1.851916	2.63E-08	4.30E-08
LINC0183	0.229212	0.878819	1.938882	0.004285	0.00497
AP002387.	3.443522	4.298544	0.319963	0.453151	0.467687
AC092134	0.387203	0.722858	0.900624	3.76E-05	5.01E-05
FGD5-AS1	10.28235	16.34959	0.669084	1.13E-09	2.10E-09
AL603839.	0.272785	0.734063	1.428142	4.54E-09	7.91E-09
MMP25-A	0.293517	0.729784	1.314025	3.40E-13	8.80E-13
ZSCAN16-	3.443201	7.500916	1.123316	1.64E-12	3.98E-12
AL133520.	0.545992	1.25653	1.202494	0.000172	0.000219
LINC0194	0.689695	1.450846	1.072865	2.74E-06	3.92E-06
FAM111A-	0.273121	0.698282	1.35427	7.48E-17	2.98E-16
RAB11B-A	6.735463	9.054312	0.426828	0.091935	0.098884
NIFK-AS1	0.570199	1.264887	1.149471	3.89E-19	2.34E-18
AC010503	12.11853	19.85186	0.71206	1.65E-05	2.25E-05
AC097468	1.646836	3.071603	0.899295	6.58E-08	1.05E-07
TMEM44-	2.565958	5.857021	1.19067	1.52E-12	3.73E-12
AL031058.	2.064092	4.936447	1.257966	3.36E-05	4.49E-05
SNHG8	19.47856	40.22252	1.046116	2.53E-11	5.47E-11
LINC0113	0.035456	0.881146	4.635298	0.044866	0.049259
LINC0112	0.949325	3.414409	1.846662	2.77E-05	3.72E-05
LINC0089	0.094482	0.652229	2.787265	7.72E-11	1.60E-10
AC022784	0.246774	1.013012	2.037387	5.16E-07	7.76E-07

AC016747	1.090886	2.809532	1.36483	8.60E-27	9.34E-25
AC012306	1.033594	1.537451	0.572871	1.35E-07	2.08E-07
AC015908	3.138826	1.795199	-0.80608	1.08E-13	2.89E-13
OTUD6B-/	1.62141	3.280372	1.016611	3.27E-18	1.57E-17
HAGLR	0.011622	1.507579	7.01919	1.39E-27	3.43E-25
LINC0148	0.203283	0.553471	1.445023	1.46E-09	2.65E-09
AL355353.	1.183044	2.465145	1.059169	0.000336	0.000418
AL359513.	0.120214	0.575208	2.258478	4.98E-17	2.02E-16
AC015922	0.947342	0.744032	-0.34852	2.36E-08	3.89E-08
AL354836.	0.4877	1.49624	1.617275	2.75E-15	8.89E-15
LINC0156	1.814743	3.527348	0.958819	0.001674	0.001973
AL441992.	1.048315	3.2731	1.642585	1.85E-20	1.43E-19
AC068506	0.12093	0.753633	2.639685	0.000148	0.000189
AC093010	12.96735	14.75318	0.186142	0.783598	0.794332
AL354872.	13.07004	8.958924	-0.54487	1.45E-09	2.63E-09
MAP4K3-[	0.88901	1.258382	0.501298	5.41E-06	7.58E-06
AC096536	0.689748	0.621477	-0.15037	0.307489	0.320934
AC026304	0.456578	0.841873	0.882741	4.57E-05	6.06E-05
SNHG20	0.361795	1.288704	1.832675	3.89E-24	9.83E-23
AC010205	0.314294	0.532439	0.7605	0.539298	0.552743
PSMA3-AS	1.731198	3.242536	0.905352	5.76E-14	1.60E-13
NORAD	23.32324	35.89563	0.62204	2.03E-11	4.45E-11
LINC0088	0.43178	1.150869	1.414354	4.27E-05	5.68E-05
AC011451	0.589111	0.804602	0.449737	0.025527	0.028322
PCED1B-A	0.576564	1.027197	0.833161	0.010876	0.012439
AC005288	2.947971	5.297561	0.845606	7.18E-11	1.50E-10
FAM99B	3.77454	1.807266	-1.06249	1.03E-14	3.16E-14
AL360181.	1.310395	2.764978	1.077266	0.027017	0.029929
AC008735	0.370896	1.546773	2.060174	6.96E-16	2.47E-15
AL365181.	0.044872	0.872083	4.28057	2.53E-16	9.56E-16
LENG8-AS	0.190327	0.882615	2.213303	1.87E-24	5.13E-23
AC020915	0.384038	1.193137	1.63544	1.47E-22	1.98E-21
ATXN1-AS	0.683745	0.94911	0.473117	0.321079	0.334175
AC009065	0.169893	0.610909	1.846331	5.94E-21	5.43E-20
AC016773	0.060701	0.578569	3.252689	3.47E-25	1.39E-23
ZNF687-A	0.677052	1.868192	1.464304	4.37E-15	1.39E-14
AC092171	0.320882	3.082692	3.264074	1.15E-27	3.43E-25
AL121832.	0.779115	2.727053	1.807435	1.49E-19	9.83E-19
AC024075	0.174663	0.549903	1.654606	5.20E-10	9.98E-10
AC012510	0.287811	0.949093	1.721429	8.56E-17	3.37E-16
LINC0094	0.00615	1.555603	7.982678	1.01E-08	1.73E-08
AC099508	1.932979	0.547204	-1.82067	4.34E-19	2.53E-18
AP000593.	0.02346	1.496655	5.995414	1.27E-10	2.58E-10
LINC0068	0.125699	0.870312	2.791565	3.60E-23	5.79E-22
NNT-AS1	1.040119	1.869607	0.845987	3.22E-12	7.51E-12
PAQR9-AS	0.733071	1.318337	0.846694	0.000147	0.000188
AC135050	5.252388	7.757446	0.562608	2.09E-09	3.74E-09
AC018653	1.692855	2.085517	0.300947	0.168558	0.178445
AC012313	0.864041	1.757568	1.024408	9.24E-07	1.38E-06
LINC0022	0.002712	1.10443	8.669661	1.59E-07	2.44E-07
IDH1-AS1	0.466803	1.204681	1.367767	1.70E-09	3.08E-09
AP000759.	0.605085	1.53736	1.345246	3.18E-14	9.23E-14
AC010894	1.398068	2.003762	0.519277	0.00832	0.00956
LINC0204	0.080913	1.092878	3.755614	0.002729	0.00319
AC015849	0.184326	0.56545	1.617139	7.65E-10	1.45E-09
MALAT1	2.157594	6.152177	1.511673	1.04E-15	3.56E-15
AC111000	0.122345	1.127003	3.20347	1.78E-17	7.60E-17
AC079360	0.437079	1.417091	1.696967	0.472083	0.486548

CARD8-AS	0.605169	0.997187	0.720527	2.74E-05	3.69E-05
HDAC4-AS	0.496505	0.986847	0.991019	1.60E-06	2.35E-06
AC068473	0.419292	1.157338	1.464782	1.80E-18	9.12E-18
AL391244.	0.351646	1.049788	1.577903	1.31E-20	1.02E-19
LINC01806	0.88503	0.848277	-0.06119	0.015203	0.017178
AC084824	0.330474	0.850634	1.364002	1.71E-15	5.58E-15
AC020910	1.313037	1.837719	0.485008	0.041741	0.045896
AP001065.	3.934716	2.676942	-0.55567	2.08E-08	3.45E-08
DNAJC3-D	0.431365	1.022508	1.24513	7.99E-11	1.65E-10
EIF3J-DT	0.46915	1.015648	1.11428	7.32E-16	2.55E-15
AC127502	0.790175	1.124895	0.509546	0.015995	0.018043
AC009686	0.171282	0.670775	1.969452	7.12E-11	1.49E-10
B4GALT1-.	2.542954	1.436607	-0.82384	7.18E-15	2.23E-14
AC079305	0.147634	0.58304	1.981569	1.24E-07	1.92E-07
AC104971	0.070403	0.597349	3.084865	0.055311	0.060369
AC004816	0.145398	0.996639	2.777066	1.32E-22	1.80E-21
LINC00342	0.389269	1.131618	1.539549	2.17E-14	6.41E-14
AC023906	0.10081	0.870858	3.110793	0.000286	0.000361
AC006205	1.49399	10.23486	2.776249	0.589914	0.603785
LINC00623	0.739846	1.692711	1.194039	2.17E-11	4.72E-11
AC010883	0.422618	1.25078	1.5654	6.47E-13	1.63E-12
AL356234.	0.05398	0.826058	3.93575	7.62E-14	2.08E-13
AC132872	0.521036	1.942457	1.898427	1.87E-23	3.46E-22
AC022509	1.14189	1.832118	0.682089	0.001776	0.002089
AC007773	0.13765	0.707799	2.362333	4.93E-18	2.31E-17
LINC02241	0.000426	0.888621	11.02666	1.28E-12	3.15E-12
LINC01480	0.049733	0.881198	4.147179	3.55E-08	5.80E-08
AC005332	0.359772	1.790952	2.31557	1.06E-26	9.84E-25
AL118558.	0.591829	1.33673	1.175456	1.64E-12	3.98E-12
CD2BP2-D	1.001605	2.272652	1.182063	7.14E-12	1.63E-11
AC005920	0.45807	1.228889	1.423713	0.000375	0.000465
LINC-PINT	0.577172	0.835552	0.533728	3.18E-05	4.27E-05
AC018647	1.577923	2.409518	0.610718	1.53E-05	2.09E-05
LNCTAM3-	0.319557	0.918125	1.522618	9.60E-15	2.96E-14
AL161668.	6.377865	2.54853	-1.32341	4.74E-16	1.73E-15
LINC02062	0.20721	0.548325	1.403935	1.58E-11	3.52E-11
AC095057	0.171051	0.559146	1.708795	2.95E-12	6.95E-12
AC011700	0.23329	0.548133	1.232399	3.56E-06	5.03E-06
AC023090	0.007746	0.626512	6.337672	7.54E-09	1.31E-08
ELF3-AS1	0.433825	2.177377	2.327406	1.04E-19	6.98E-19
LINC02381	0.567336	2.29375	2.015431	0.993627	0.993627
AL355574.	0.219373	0.91761	2.064496	6.62E-19	3.63E-18
ENTPD3-A	1.189089	1.69434	0.510867	5.41E-05	7.15E-05
ITIH4-AS1	0.67685	1.062676	0.650793	0.022973	0.025641
AL161669.	0.434697	0.777052	0.837999	0.143659	0.152961
AL031722.	1.244404	0.903098	-0.4625	1.91E-06	2.78E-06
AC107375	0.162585	0.732007	2.170663	9.91E-26	5.24E-24
AC010761	0.219843	0.749769	1.76997	8.80E-18	3.97E-17
AC011468	0.290868	1.169254	2.007151	8.44E-19	4.46E-18
AL365181.	0.226268	4.241738	4.22855	7.58E-18	3.44E-17
ASH1L-AS	0.294663	0.774384	1.393984	5.26E-18	2.45E-17
AP000254.	1.254763	2.521725	1.006996	6.47E-13	1.63E-12
AL449106.	0.405331	0.751936	0.891509	0.021328	0.023913
AC018521	0.699756	1.101141	0.654076	0.000765	0.000919
AL031186.	0.123074	0.595767	2.275221	4.87E-21	4.68E-20
AC068580	0.204722	0.892079	2.123503	2.97E-13	7.73E-13
AC034231	0.254385	0.768437	1.594914	2.31E-15	7.51E-15
LINC00261	37.5636	22.38916	-0.74654	4.56E-12	1.06E-11

FZD4-DT	0.515213	0.655907	0.348321	0.970104	0.971417
MAFG-DT	0.119233	1.476454	3.630285	7.98E-25	2.57E-23
AC002398	0.309954	0.894128	1.528426	4.96E-15	1.57E-14
MIR4435-:	0.372212	2.014476	2.436208	9.95E-23	1.42E-21
PSORS1C3	0.144938	0.911937	2.653502	1.35E-09	2.48E-09
AC013275	0.098836	0.983149	3.3143	1.05E-07	1.64E-07
AC068888	0.742697	1.509609	1.023329	1.96E-10	3.89E-10
ELFN1-AS.	0.04566	0.910602	4.317824	0.005869	0.006775
ARHGAP2	0.177379	0.584585	1.720578	8.63E-21	7.17E-20
AC073896	3.202745	6.253201	0.965286	6.92E-10	1.32E-09
AC022424	0.008662	0.788276	6.507832	1.47E-07	2.27E-07
HOTAIRM:	0.449897	0.66508	0.563934	0.16479	0.174706
AC015813	0.209155	0.796465	1.929041	6.08E-17	2.44E-16
LINC0154	0.128305	2.128594	4.052255	0.00056	0.000681
LIVAR	3.427433	5.415685	0.660016	0.201484	0.211787
AC025265	0.154723	0.565949	1.870984	4.97E-12	1.15E-11
RNASEH1-	1.306506	2.769461	1.083891	9.79E-16	3.38E-15
AC019117	0.246056	0.899713	1.870478	0.000429	0.000527
PTOV1-AS	0.473182	1.231985	1.380518	4.84E-23	7.62E-22
MIR3936H	0.380139	0.679116	0.837132	2.62E-06	3.77E-06
LINC0084	2.098642	4.264081	1.022779	3.39E-16	1.26E-15
AC004148	0.262708	0.790841	1.589929	1.07E-15	3.65E-15
AL359504.	0.175786	0.544795	1.631892	6.39E-14	1.76E-13
AC018904	0.950887	2.41173	1.342723	4.94E-16	1.79E-15
AC007405	0.903436	2.434416	1.430082	8.72E-15	2.70E-14
AGAP2-AS	0.705561	1.354837	0.941276	0.273315	0.286072
AL035461.	0.370123	2.059544	2.476249	6.52E-20	4.55E-19
AC142472	0.218401	0.663329	1.602746	5.37E-19	3.03E-18
AC020978	4.697383	2.171596	-1.1131	1.64E-15	5.38E-15
MROCKI	0.434821	0.801447	0.882185	0.024808	0.027606
AC040977	1.058995	3.028163	1.515747	3.00E-18	1.45E-17
AL645933.	0.712656	1.56922	1.13877	1.98E-08	3.29E-08
RUSC1-AS	0.286733	1.654775	2.528857	1.14E-24	3.38E-23
AC126614	0.605604	0.578113	-0.06702	0.001516	0.001795
LINC0260	0.686024	1.567989	1.192585	3.43E-13	8.85E-13
AC087741	0.352441	1.30265	1.885998	1.72E-13	4.54E-13
AC009005	0.189461	1.413915	2.899726	3.69E-20	2.70E-19
TUSC8	1.358903	2.506884	0.883452	0.141826	0.151445
AC015871	0.359205	0.808541	1.170513	5.86E-13	1.49E-12
AL035446.	0.18242	1.69007	3.211748	0.060196	0.065507
LINC0095	0.319173	0.731721	1.196957	3.95E-08	6.42E-08
SNHG16	1.877126	2.991371	0.672282	2.89E-06	4.12E-06
AFDN-DT	0.31699	0.691875	1.126076	1.38E-09	2.53E-09
AL604028.	1.679746	2.619729	0.641175	2.05E-11	4.48E-11
AL135999.	0.165959	0.647764	1.964643	1.89E-17	7.92E-17
AC138207	1.237481	1.657138	0.421288	0.153867	0.163595
AC108673	1.687319	2.392887	0.504019	0.015205	0.017178
AL359076.	2.407019	1.953361	-0.30129	5.19E-06	7.29E-06
TMPO-AS	0.223928	1.154242	2.365841	1.34E-23	2.75E-22
AC069281	0.55303	0.90158	0.705098	7.29E-08	1.16E-07
AC016394	0.189738	0.83029	2.129609	8.24E-21	7.09E-20
TAF1A-AS	0.747174	1.499026	1.004508	1.23E-09	2.28E-09
AC024075	0.80442	2.408968	1.582394	4.93E-21	4.68E-20
AC009275	0.087308	0.733148	3.069928	1.02E-08	1.74E-08
LINC0023	0.10789	0.636681	2.561005	1.43E-05	1.96E-05
AC025857	2.963626	4.664359	0.654316	0.053479	0.058456
KCNMB2-.	0.017109	0.749221	5.452522	1.26E-14	3.84E-14
BX284668.	3.892017	8.167327	1.069346	1.26E-08	2.14E-08

AC011477	0.542014	1.39663	1.365547	2.86E-09	5.10E-09
FAM245A	0.588299	0.532543	-0.14365	1.77E-07	2.70E-07
AC078993	0.026049	0.834589	5.001779	3.55E-10	6.90E-10
NEAT1	5.481795	12.6457	1.205927	9.87E-09	1.70E-08
SCAT8	0.273798	1.038534	1.923365	3.09E-05	4.15E-05
AP003174.	0.086242	0.559118	2.69669	1.90E-11	4.19E-11
AL365330.	0.376062	0.925295	1.298943	1.51E-11	3.39E-11
AC007541	0.39944	1.043334	1.385149	1.04E-15	3.56E-15
AC005332	5.082137	8.07039	0.667203	1.08E-10	2.21E-10
AL021578.	0.686155	0.594479	-0.20691	0.103365	0.110855
AC020765	0.257708	0.923857	1.841933	4.11E-19	2.44E-18
AP000355.	1.857473	3.721001	1.00235	0.087502	0.09441
LINC0066	0.119604	1.405468	3.554708	1.62E-10	3.23E-10
LINC0153	0.326395	0.746582	1.193683	2.13E-12	5.12E-12
LINC0176	17.83771	8.809328	-1.01783	2.66E-12	6.34E-12
AC016888	2.416702	6.058208	1.325851	2.70E-12	6.41E-12
HCP5	3.080273	8.09691	1.394313	1.13E-09	2.11E-09
AL031985.	0.218939	0.7233	1.724069	8.18E-20	5.55E-19
AL606489.	0.055938	0.834341	3.898725	8.23E-24	1.84E-22
SNHG3	0.633349	3.314609	2.387767	3.52E-23	5.78E-22
HEIH	5.341754	12.86642	1.268225	2.07E-24	5.47E-23
LBX2-AS1	8.645909	13.48136	0.640876	1.19E-05	1.64E-05
LMNTD2-/	0.195997	0.824573	2.072817	7.63E-14	2.08E-13
AC068580	0.311359	1.017235	1.708003	3.09E-14	9.04E-14
PRRT3-AS	0.442701	1.748381	1.981615	2.87E-10	5.64E-10
LINC0249	5.231562	4.44856	-0.2339	0.000684	0.000826
LINC0137	6.671672	4.610748	-0.53305	2.43E-06	3.50E-06
AC005332	0.232729	0.563755	1.276415	1.78E-11	3.94E-11
AP002360.	1.214584	4.067104	1.743539	8.08E-19	4.33E-18
LINC0084	36.99786	21.28071	-0.7979	3.09E-12	7.24E-12
AC011472	0.397519	0.692401	0.800582	9.94E-05	0.000129
SNHG10	0.455102	1.518934	1.738798	1.56E-23	2.99E-22
AC036176	3.679289	2.901494	-0.34263	1.28E-05	1.76E-05
AC093227	0.481862	0.856739	0.830237	1.56E-06	2.28E-06
AC087741	0.18484	0.716305	1.954298	6.13E-19	3.41E-18
AC018645	1.575443	2.806275	0.832898	1.44E-09	2.62E-09
U62317.2	1.545934	3.76831	1.285439	5.74E-16	2.06E-15
NRAV	0.820404	2.090722	1.349595	3.98E-19	2.37E-18
AC106876	1.679182	2.336199	0.476404	0.052275	0.057224
MAGI2-AS	1.564102	0.758725	-1.04369	1.16E-13	3.10E-13
LINC0282	0.01707	0.838233	5.617827	7.33E-05	9.62E-05
SLC6A1-A	0.151585	0.554465	1.87097	4.88E-06	6.87E-06
AC139530	0.504627	1.532106	1.602227	9.25E-21	7.52E-20
AC106820	0.182074	0.936768	2.363168	6.44E-19	3.56E-18
VPS9D1-A	0.293666	0.823905	1.488303	1.16E-06	1.71E-06
AC009171	0.396748	0.766655	0.950352	3.56E-06	5.03E-06
AP001372.	0.554063	1.199578	1.114406	1.22E-15	4.10E-15
AC009974	1.175395	1.406143	0.258598	0.789268	0.798986
AL355488.	0.159005	0.990814	2.639539	9.07E-25	2.80E-23
AC055822	0.22162	0.704284	1.66807	4.86E-14	1.37E-13
SNHG1	1.243861	6.041754	2.280142	4.47E-26	3.01E-24
AC040970	0.106725	0.629852	2.561114	7.47E-06	1.04E-05
ZNF793-A	0.1002	0.571155	2.510992	0.000598	0.000725
MELTF-AS	0.112246	0.961025	3.097909	7.08E-21	6.24E-20
AP000894.	0.374369	1.259622	1.75046	3.26E-13	8.48E-13
SIAH2-AS	0.406629	0.604561	0.572176	0.929989	0.935043
AC012379	0.998014	0.768	-0.37795	3.07E-11	6.60E-11
SNHG7	1.412275	6.609583	2.226538	1.87E-24	5.13E-23

CRIM1-DT	0.851886	3.148073	1.885737	0.000112	0.000144
WNT5A-A	0.191036	0.608341	1.671033	0.003879	0.004507
AL392172.	1.84866	5.774383	1.643187	2.30E-21	2.50E-20
SNHG12	0.473602	1.811267	1.935252	7.10E-22	8.62E-21
C1RL-AS1	0.68548	1.195789	0.802776	0.000298	0.000374
AC104794	1.431963	3.121834	1.124399	4.55E-14	1.29E-13
AC027796	0.106294	0.583389	2.456399	1.25E-17	5.51E-17
ZNF503-A	0.411787	1.040383	1.337146	1.67E-17	7.18E-17
WAC-AS1	2.956688	5.839313	0.981816	7.17E-16	2.53E-15
AC090587	0.486699	1.009292	1.052243	4.96E-08	8.01E-08
LINC02362	3.932492	2.495549	-0.65609	9.87E-09	1.70E-08
PTOV1-AS	0.453243	1.55688	1.780301	2.66E-18	1.30E-17
AC067838	0.515352	0.869123	0.754002	0.000372	0.000461
AL035661.	4.10745	2.72663	-0.59112	1.18E-10	2.41E-10
LINC02754	2.39994	1.436792	-0.74015	1.70E-10	3.38E-10
MHENCR	1.64299	4.408032	1.42381	4.83E-17	1.96E-16
JPX	1.936294	3.054207	0.6575	2.24E-10	4.42E-10
F11-AS1	3.343398	2.224688	-0.58771	4.68E-10	9.03E-10
AC125257	1.260418	2.688782	1.093051	1.38E-21	1.53E-20
LINC01146	5.625523	4.69154	-0.26193	0.001633	0.00193
AL353622.	0.247943	0.804718	1.698475	1.13E-14	3.46E-14
TMEM220-	3.700262	2.085687	-0.8271	2.13E-14	6.34E-14
TP53TG1	16.87929	21.69633	0.362197	0.142163	0.151586
AL139246.	0.874657	1.518877	0.796216	0.008411	0.009649
LINC00513	0.157961	0.647675	2.035702	5.61E-07	8.38E-07
BAIAP2-DT	1.719264	4.877977	1.504492	8.92E-19	4.68E-18
SCGB1B2P	0.394358	0.774968	0.974633	2.44E-05	3.30E-05
AC092171	0.415698	1.573045	1.919952	1.35E-17	5.91E-17
APTR	1.110512	2.650322	1.254943	1.32E-22	1.80E-21
AL162586.	0.329785	0.641546	0.960025	4.45E-05	5.92E-05
AC096677	1.265095	1.223342	-0.04842	0.014155	0.016041
AC099684	2.683282	4.204931	0.648083	0.381965	0.395875
GPRC5D-A	0.401211	0.934751	1.220223	1.66E-19	1.07E-18
PRANCR	0.415161	1.046368	1.333649	9.09E-18	4.08E-17
AC023157	0.545236	1.186093	1.121264	1.01E-09	1.90E-09
LINC02027	5.577541	1.867271	-1.5787	1.67E-19	1.07E-18
AC000123	0.433409	0.95974	1.146914	1.65E-12	4.00E-12
AC005586	0.251212	0.582299	1.212852	1.09E-06	1.62E-06
ZNF213-A	0.499615	1.267142	1.342689	9.14E-21	7.52E-20
AC009237	0.246232	0.665483	1.434382	5.71E-12	1.31E-11
DNMBP-A	0.321283	0.590299	0.877604	0.665801	0.677707
U62317.1	0.259178	0.800768	1.627444	1.35E-08	2.27E-08
FOXD2-AS	0.225472	1.579585	2.808525	5.05E-21	4.73E-20
C2CD4D-A	1.028418	4.638335	2.17318	5.25E-19	2.99E-18
AC116025	1.003303	0.725564	-0.46758	1.23E-06	1.82E-06
AC131009	0.4517	1.221894	1.435683	1.75E-12	4.23E-12
AC026462	1.320374	3.73676	1.500841	5.91E-05	7.79E-05
LINC00987	1.213899	1.105047	-0.13554	1.81E-06	2.64E-06
AC068987	0.268475	1.605063	2.57977	5.60E-14	1.57E-13
AC020916	1.443022	2.052602	0.508361	0.038578	0.042482
AC090589	0.359482	0.774625	1.107579	5.67E-08	9.15E-08
AC105105	3.523956	2.575652	-0.45226	8.75E-05	0.000114
YTHDF3-A	0.868331	2.105716	1.277994	1.34E-10	2.70E-10
AP003486.	0.341558	0.827403	1.276461	1.61E-15	5.33E-15
AC080129	0.121195	0.789454	2.703525	1.79E-17	7.61E-17
AJ009632.1	0.319346	1.330368	2.058634	0.020947	0.023521
AL133419.	0.755922	0.616137	-0.29499	9.47E-07	1.41E-06
AC074212	0.153294	0.633311	2.046613	7.80E-23	1.15E-21

SNHG5	13.08592	23.18405	0.825117	0.001457	0.001728
AP002807.	0.172393	0.861442	2.321051	3.65E-18	1.74E-17
AC103760	1.523698	2.618749	0.781301	0.000762	0.000917
AC132192	0.117529	0.973848	3.050686	4.78E-28	3.43E-25
NRSN2-AS	0.361432	0.952883	1.398574	4.25E-12	9.90E-12
LINC0178€	0.300857	0.910318	1.597294	8.38E-14	2.26E-13
AC012313	0.609874	1.304765	1.097206	9.70E-17	3.78E-16
GAPLINC	0.071529	0.893981	3.643653	4.51E-14	1.28E-13
ZNF674-A	0.707609	1.038335	0.553249	2.41E-05	3.28E-05
AP001318.	1.620166	2.361194	0.543375	2.92E-07	4.41E-07
LINC01671	0.162633	0.682322	2.068836	0.021908	0.024526
AC090152	0.227408	0.689528	1.60033	0.950517	0.953093
AL117379.	0.126153	0.619514	2.295958	1.10E-21	1.24E-20
BACE1-AS	0.560946	2.149493	1.938062	5.04E-25	1.78E-23
PDCD4-AS	0.741459	1.135477	0.61486	0.000255	0.000322
AP006216.	2.828866	1.545986	-0.8717	1.67E-11	3.71E-11
LUCAT1	0.021123	0.578824	4.776251	1.12E-08	1.91E-08
RAD51-AS	0.343281	1.056517	1.621855	7.47E-20	5.16E-19
HORMAD2	2.360755	4.711129	0.996824	0.940706	0.944535
AL138724.	0.541524	0.918549	0.762332	3.45E-05	4.61E-05
AC060766	0.377714	0.66814	0.822856	0.000476	0.000582
AC006026	0.2707	0.648452	1.260303	0.001284	0.001528
AC090164	0.076786	0.727029	3.243102	7.20E-14	1.98E-13
AL844908.	0.221688	0.550804	1.313007	1.09E-07	1.71E-07
AC026979	1.295604	2.023229	0.643035	0.087521	0.09441
AC046143	0.469098	0.88241	0.91156	0.000277	0.000349
AP000424.	0.236931	0.728968	1.621391	0.090492	0.097473
AC079466	0.038081	4.695623	6.946087	1.47E-06	2.17E-06
USP46-AS	0.623257	1.103975	0.824809	7.88E-09	1.37E-08
CR936218.	0.312461	0.861487	1.463154	0.001408	0.001673
AC007406	0.16021	1.189949	2.892865	1.98E-06	2.85E-06
AC012640	1.966903	2.428885	0.304368	0.42268	0.436848
AP003555.	0.732457	0.683407	-0.1	9.12E-06	1.26E-05
TMEM147	0.183058	0.685395	1.904638	3.38E-22	4.31E-21
CEBPA-DT	1.43906	4.884097	1.762965	3.55E-15	1.14E-14
AC021078	0.380296	0.829084	1.124397	9.11E-14	2.45E-13
AL162595.	0.249471	0.77042	1.626774	1.36E-17	5.93E-17
AC005229	0.660627	1.681631	1.347953	1.86E-19	1.19E-18
AC027307	2.892197	8.621631	1.575795	4.19E-24	1.00E-22
MID1IP1-A	0.322096	1.010146	1.649002	2.55E-09	4.56E-09
AC008608	0.970355	2.129741	1.134093	1.73E-09	3.12E-09
AC104534	0.124183	0.870038	2.80861	5.88E-11	1.26E-10
PAXIP1-AS	0.820628	1.189212	0.535206	0.000538	0.000657
AL139287.	0.856955	2.656586	1.632282	2.35E-21	2.52E-20
AC010973	0.110305	0.626642	2.506145	7.06E-26	4.35E-24
AC090198	0.640918	0.821594	0.358285	0.077539	0.083887
AC022144	0.523767	1.218033	1.217555	0.000648	0.000785
AC080112	1.838957	3.544	0.946491	7.88E-13	1.96E-12
KMT2E-AS	2.387686	5.900016	1.305106	1.52E-13	4.04E-13
AP006621.	0.24696	1.609354	2.704131	1.54E-16	5.85E-16
AC005261	1.477345	2.973788	1.009295	9.39E-18	4.16E-17
ZEB1-AS1	0.274888	0.805408	1.550878	3.63E-22	4.56E-21
RAB30-DT	1.130121	2.478681	1.133095	2.14E-18	1.07E-17
CASC19	0.646149	1.094123	0.759837	0.36043	0.374605
AC007038	0.177519	0.604213	1.767087	3.11E-19	1.90E-18
AC083809	0.105745	2.638211	4.640902	0.665007	0.677707
AC005261	1.635385	3.93295	1.265981	2.65E-23	4.56E-22
AC115619	37.88454	26.16042	-0.53422	3.50E-09	6.17E-09

MIR4458H	0.66621	1.560526	1.227984	1.95E-09	3.50E-09
AC006449	0.636535	1.282471	1.010615	1.33E-09	2.45E-09
AP000757.	0.044953	0.670269	3.898239	0.106899	0.114479
AL390728.	0.99226	3.999798	2.011137	1.78E-18	9.08E-18
AL591895.	2.803406	10.15937	1.857558	3.09E-21	3.22E-20
LINC0143(	0.088615	1.690845	4.254042	5.70E-06	7.98E-06
AC004012	0.364653	0.650214	0.834389	0.602755	0.616075
MUC20-O	0.327767	0.84424	1.364981	5.80E-18	2.66E-17
LINC0099	0.581848	1.178157	1.017817	8.01E-16	2.78E-15
STPG3-AS	0.139222	0.77431	2.475524	2.17E-14	6.41E-14
LINC0114	0.429128	0.766701	0.837255	0.000118	0.000152
AC145343	0.145612	0.786465	2.433257	5.73E-18	2.65E-17
AL023284.	3.66919	4.758324	0.374992	0.707368	0.719028
AP005482.	0.704335	1.114518	0.662086	7.54E-08	1.19E-07
AC015917	3.265143	5.102394	0.644028	0.098514	0.105806
AC004918	0.394433	1.168577	1.5669	4.77E-18	2.25E-17
AC108134	0.14337	0.57769	2.010556	7.32E-14	2.00E-13
AC253536	0.418573	1.685762	2.00985	3.37E-09	5.96E-09
LINC0118(	0.076483	0.778008	3.346572	1.61E-14	4.83E-14
AC024060	0.86913	2.733276	1.652988	8.19E-23	1.19E-21
SNHG17	1.42856	3.703286	1.374244	3.19E-16	1.19E-15
SBF2-AS1	0.233662	0.755556	1.693116	3.38E-22	4.31E-21
SNHG21	0.193549	0.642201	1.730323	6.92E-21	6.17E-20
AC079922	0.431565	0.885701	1.037243	4.72E-13	1.20E-12
LINC0242	10.75337	4.042471	-1.41148	4.04E-17	1.67E-16
ALDH1L1-	0.319396	0.689547	1.110303	0.003118	0.003628
AC007996	0.225625	0.713521	1.661032	1.58E-15	5.24E-15
Z98257.1	0.170202	0.806715	2.244809	1.08E-08	1.84E-08
AGAP1-IT	1.670174	1.127569	-0.56678	1.67E-08	2.79E-08
AC243964	0.765912	1.811367	1.241828	1.96E-06	2.84E-06
ZBED5-AS	3.092863	4.968219	0.683786	1.24E-07	1.92E-07
LINC0108	0.248189	1.233953	2.313775	3.82E-26	2.83E-24
AC008915	0.92107	1.967004	1.094618	1.56E-15	5.22E-15
AL360181.	0.334574	0.846639	1.33942	5.23E-10	9.99E-10
LINC0018	0.093601	0.619327	2.726101	0.06811	0.073902
AC139100	0.144009	0.980064	2.766721	6.93E-22	8.55E-21
LINC0203	2.501451	1.425327	-0.81147	6.65E-11	1.40E-10
SNHG32	9.754471	36.20809	1.892177	3.75E-25	1.39E-23
DSCR8	0.002448	1.188885	8.924002	8.02E-09	1.39E-08
AL021707.	0.447574	1.213484	1.438958	5.47E-12	1.26E-11
AL022328.	0.734639	2.099015	1.514605	6.21E-17	2.48E-16
AC026401	0.913817	3.270511	1.839539	2.24E-18	1.11E-17
PRR26	0.766131	0.679338	-0.17346	1.31E-05	1.79E-05
SNHG9	3.260921	9.528414	1.546957	1.33E-10	2.69E-10
LINC0117	0.43653	1.210682	1.471667	1.29E-15	4.34E-15
