# Supplementary material for: Construction of a novel immune-related lncRNA signature and its potential to predict the immune status of patients with hepatocellular carcinoma
Source: BMC Cancer. 2021 Dec 19;21:1347. doi: 10.1186/s12885-021-09059-x (PMC8684648; doi:10.1186/s12885-021-09059-x)
Supplement: Supplementary file 2 — Additional file 2. [file 12885_2021_9059_MOESM2_ESM.pdf]

gene	conMean	treatMean	logFC	pValue	fdr
AC093673	3.163426	7.33399	1.21311	2.10E-11	4.58E-11
AC092535	1.036945	6.471465	2.641754	7.00E-19	3.81E-18
AC099850	0.183166	2.081554	3.506435	3.81E-18	1.81E-17
U91328.1	0.472912	1.228562	1.377327	2.13E-14	6.34E-14
LINC0010€	0.37402	1.6431	2.135232	1.20E-15	4.04E-15
PPP1R14B	0.292719	1.214102	2.0523	1.39E-14	4.20E-14
SNHG6	11.70091	45.53758	1.960437	3.16E-21	3.25E-20
AC109322	0.245663	1.545494	2.653313	1.87E-27	3.47E-25
FAM99A	13.79846	5.52311	-1.32095	7.91E-14	2.14E-13
AL359921.	0.474474	1.734024	1.869724	1.10E-20	8.76E-20
AC007566	0.222305	0.549459	1.305468	4.23E-09	7.41E-09
AC005840	0.281507	0.568707	1.014511	1.34E-08	2.27E-08
COA6-AS:	1.036828	3.322924	1.680276	7.34E-18	3.35E-17
AC007099	0.013203	0.612756	5.536428	1.02E-07	1.59E-07
LINC0113ε	0.226353	1.055284	2.220986	5.96E-23	9.19E-22
TYMSOS	0.779702	2.927928	1.908885	3.63E-14	1.05E-13
AC010735	0.395278	1.056607	1.4185	1.02E-09	1.91E-09
AC084018	0.231808	1.102952	2.250367	9.36E-17	3.67E-16
UBR5-AS1	0.675682	1.83851	1.444121	1.57E-23	2.99E-22
AC124798	0.181986	0.61745	1.762497	0.025528	0.028322
SH3BP5-A	0.322053	0.662067	1.039677	1.34E-09	2.47E-09
AL021807.	0.07637	0.629068	3.042135	1.32E-16	5.08E-16
THUMPD3	0.417553	1.434058	1.780072	6.85E-25	2.30E-23
AC010531	0.378412	0.970835	1.359269	1.94E-06	2.81E-06
AC016405	0.210645	0.929251	2.141253	3.03E-12	7.11E-12
LINC0229ε	0.129878	0.703709	2.437816	8.98E-05	0.000117
AC012615	0.456284	1.737743	1.929209	1.10E-23	2.32E-22
AC023043	0.252395	0.890745	1.81933	6.65E-13	1.67E-12
AL109615.	0.045131	0.733589	4.022782	1.82E-11	4.01E-11
SNHG19	10.30831	26.1105	1.340823	1.57E-06	2.29E-06
AP001505.	1.278712	3.855314	1.592157	1.77E-12	4.27E-12
AL365203.	0.682079	2.228084	1.707792	3.48E-15	1.12E-14
MAPKAPK	1.350742	3.967567	1.554503	8.83E-27	9.34E-25
AC007292	0.289337	1.002109	1.792216	4.50E-19	2.60E-18
TNFRSF14	0.383856	1.040593	1.438768	1.70E-06	2.47E-06
AC005899	0.189086	0.599536	1.664806	4.92E-10	9.45E-10
AL391422.	1.193266	2.507501	1.071334	1.53E-11	3.41E-11
AL050341.	0.901121	2.179595	1.274267	7.24E-16	2.54E-15
AC064836	0.488655	1.086648	1.152998	7.08E-10	1.34E-09
AL355802.	0.122382	0.583838	2.254182	5.07E-20	3.64E-19
ZFAS1	6.45919	19.53775	1.596839	1.38E-17	5.96E-17
MUC12-A:	0.061443	0.620857	3.336938	0.000339	0.000422
AC091729	1.120439	2.851027	1.347418	9.19E-18	4.10E-17
FLVCR1-D	0.231485	1.111709	2.263791	2.61E-19	1.61E-18
POLR2J4	0.618527	1.265251	1.032515	1.01E-16	3.92E-16
SLC25A25-	0.693352	1.842662	1.410132	8.60E-10	1.62E-09
AC073611	0.204749	0.733007	1.839972	1.15E-18	5.94E-18
ASB16-AS	0.835192	2.624523	1.651876	2.86E-25	1.25E-23
AL031673.	0.495194	1.843383	1.896291	2.06E-19	1.30E-18
AC008764	0.420982	1.184437	1.492371	8.15E-11	1.68E-10
BX322562.	0.793129	2.019644	1.348473	0.016542	0.018631
AL358472.	0.389859	0.892932	1.195597	4.37E-16	1.61E-15
AC008736	0.374566	1.090306	1.541439	2.29E-12	5.48E-12
TRIM52-A	2.198441	4.953459	1.171956	1.09E-15	3.71E-15
AC016394	0.13097	0.719759	2.45828	1.09E-25	5.35E-24
LINC0150:	0.556853	1.431528	1.362188	0.002721	0.003186
AC009779	1.793328	3.783868	1.077222	1.42E-16	5.45E-16

PXN-AS1	0.720148	1.894678	1.395587	4.65E-21	4.59E-20
PRR34-AS	0.935418	3.428075	1.873715	2.02E-13	5.28E-13
PVT1	0.118649	0.929567	2.969859	2.90E-20	2.15E-19
SNHG25	0.651616	5.018619	2.945195	1.99E-23	3.60E-22
AC004687	0.196521	0.638455	1.699901	1.53E-06	2.25E-06
ZFPM2-AS	0.053865	1.493454	4.793167	1.20E-08	2.05E-08
AC104825	0.409677	0.974514	1.250195	1.07E-10	2.19E-10
AC009407	3.991088	10.44651	1.388166	3.63E-09	6.39E-09
AC103691	0.443405	0.929335	1.067575	8.70E-08	1.37E-07
AL390198.	0.425944	1.27319	1.579712	0.000142	0.000181
PITPNA-A	1.65081	4.433619	1.425311	6.23E-16	2.23E-15
LINC0029	0.862224	1.874373	1.120273	2.84E-18	1.38E-17
AL162582.	0.133943	1.827624	3.770279	1.23E-09	2.28E-09
AC010280	3.545021	0.881215	-2.00823	3.36E-21	3.41E-20
AL160006.	0.215474	0.817242	1.923247	7.63E-22	8.96E-21
AC010969	0.527831	1.193937	1.177578	1.85E-17	7.84E-17
AC010542	0.23622	0.873335	1.886403	1.17E-18	6.03E-18
AL021328.	1.38585	0.473786	-1.54846	1.47E-16	5.60E-16
AC009283	1.814618	4.569689	1.332431	7.56E-19	4.08E-18
AC244090	3.362677	9.028119	1.424815	1.98E-17	8.21E-17
AC008267	2.82838	6.126457	1.115077	6.67E-11	1.40E-10
AC147067	0.332911	0.811313	1.285123	1.07E-05	1.48E-05
AC009237	1.338443	3.017227	1.172668	1.64E-15	5.38E-15
LINC0177	0.756309	1.989617	1.395443	2.32E-07	3.52E-07
AC002456	0.52978	1.384336	1.385728	8.84E-05	0.000115
AC136475	0.532915	1.350711	1.341741	7.27E-13	1.82E-12
AC138696	0.58664	2.541744	2.11527	2.28E-20	1.72E-19
AC008443	0.643931	1.837001	1.512374	4.13E-10	7.98E-10
SERTAD4-	0.052423	0.667172	3.66979	5.39E-16	1.94E-15
AC132872	0.248568	0.632864	1.348257	1.93E-09	3.48E-09
AC084036	1.128049	3.565437	1.660249	4.51E-16	1.65E-15
CTBP1-DT	0.738001	1.797603	1.28438	1.00E-21	1.16E-20
AL162413.	0.01103	4.245629	8.588431	3.05E-09	5.42E-09
AL121944.	1.261878	2.527907	1.002371	6.35E-11	1.35E-10
AC007448	0.264069	0.772098	1.547867	1.03E-09	1.94E-09
BX537318.	0.477849	1.423758	1.575077	6.92E-21	6.17E-20
ARRDC1-A	1.527505	3.569038	1.224358	8.45E-24	1.84E-22
LINC0142	0.074323	0.835773	3.491224	8.19E-12	1.87E-11
AL445524.	1.830088	12.66058	2.790358	8.48E-26	4.82E-24
DLG5-AS1	0.179582	1.385736	2.947938	1.56E-23	2.99E-22
DLGAP1-A	1.271828	3.068002	1.270396	2.09E-10	4.13E-10
AL049840.	2.057305	5.375175	1.385556	1.63E-19	1.07E-18
CYTOR	0.722748	4.130732	2.514832	8.43E-21	7.16E-20
AC011445	0.303189	2.805598	3.210017	8.52E-21	7.16E-20
AC110285	0.228376	1.539482	2.752965	3.56E-19	2.16E-18
AC083880	0.202429	0.676264	1.740175	1.55E-14	4.67E-14
MINCR	0.769633	3.339484	2.117382	5.23E-21	4.84E-20
AL023803.	0.581269	1.525726	1.392218	2.43E-05	3.28E-05
AL928654.	0.307821	0.91663	1.574249	9.11E-12	2.07E-11
LINC0131	0.163606	0.665645	2.024525	4.72E-13	1.20E-12
AP000424.	0.121326	0.591554	2.285618	0.001875	0.002199
AC022150	0.203157	0.547938	1.431416	0.000317	0.000395
CHKB-DT	0.521489	1.286361	1.302586	2.63E-16	9.88E-16
AC006504	0.201836	0.729382	1.853491	7.10E-16	2.51E-15
AC006213	0.253245	0.839264	1.728594	3.29E-10	6.43E-10
PRKAR1B-	0.312737	0.842778	1.430202	1.88E-08	3.14E-08
AC127024	0.187299	0.878004	2.228885	9.47E-21	7.62E-20
AC026471	0.700185	1.612986	1.203926	3.73E-14	1.07E-13

MIR210HG	0.439851	1.419384	1.69018	0.000416	0.000512
AC009065	0.518518	1.219769	1.234141	5.97E-15	1.87E-14
AL359643.	0.336933	0.836039	1.311108	3.18E-14	9.23E-14
AC136475	0.440161	1.324741	1.589607	0.011424	0.013046
AL118516.	1.739653	3.558272	1.032377	2.12E-10	4.19E-10
AL158071.	0.203916	0.619443	1.602999	3.73E-10	7.22E-10
AL022328.	0.317449	0.790558	1.316347	1.97E-11	4.32E-11
AFAP1-AS	0.005129	1.323426	8.011325	1.66E-13	4.40E-13
SNHG11	1.670991	5.161074	1.626967	3.39E-23	5.70E-22
LINC0020	0.289346	1.405556	2.280276	1.03E-21	1.17E-20
AC010331	0.119088	0.606362	2.348153	7.63E-22	8.96E-21
NCK1-DT	0.636228	1.416334	1.154545	2.25E-19	1.41E-18
AC011477	0.378295	0.986702	1.383104	2.25E-07	3.42E-07
AL354892.	1.056558	2.284709	1.112638	8.24E-08	1.30E-07
AC091271	0.670871	1.479532	1.141033	2.30E-08	3.79E-08
AC012467	0.571004	1.269549	1.152742	5.50E-20	3.91E-19
AP000240.	0.213293	0.639438	1.583968	1.17E-09	2.17E-09
Z95115.1	0.339388	1.009343	1.572409	5.82E-14	1.61E-13
SNHG30	1.354942	3.944495	1.541609	8.44E-19	4.46E-18
GIHCG	0.38911	1.925016	2.306622	3.99E-24	9.83E-23
TBX2-AS1	0.171174	0.777199	2.182823	9.24E-12	2.08E-11
AC104958	3.574543	12.10274	1.759503	3.24E-14	9.37E-14
AC103706	0.254451	1.342626	2.399596	7.37E-21	6.42E-20
AL132989.	0.295301	0.66163	1.163838	1.61E-07	2.47E-07
AC005586	0.291505	0.867054	1.572601	8.31E-13	2.06E-12
ST7-AS1	0.206386	0.545946	1.403417	9.95E-11	2.05E-10
AC012409	1.705277	0.83098	-1.03712	2.68E-12	6.37E-12
AC104113	0.486284	1.070232	1.138052	9.24E-12	2.08E-11
AC015982	0.464077	0.946198	1.027776	1.55E-10	3.11E-10
RPARP-AS	0.721191	1.489844	1.046709	4.39E-14	1.25E-13
CCDC18-A	0.248727	1.024184	2.041839	6.26E-23	9.45E-22
AC007114	1.295318	2.730659	1.075942	6.19E-11	1.32E-10
AC015912	0.368842	1.482856	2.007305	7.30E-11	1.52E-10
CAMTA1-I	0.242566	0.593918	1.291887	1.16E-07	1.81E-07
AC026369	0.198162	0.548709	1.469362	1.89E-13	4.97E-13
TAT-AS1	0.427066	0.881869	1.046106	0.009454	0.010829
AC092490	0.053888	0.711839	3.723513	9.73E-05	0.000127
AP002748.	0.545956	1.294132	1.245128	2.48E-14	7.29E-14
SSTR5-AS	0.009849	1.461736	7.213482	0.000309	0.000387
ITGB1-DT	0.566653	1.563889	1.464602	0.00106	0.001267
MAP3K2-D	0.419095	1.008296	1.266572	1.30E-09	2.40E-09
AL354733.	0.26252	0.553279	1.07558	0.001074	0.001282
ANKRD10-	0.853368	2.269042	1.410844	1.66E-07	2.54E-07
AC005696	0.36597	0.830937	1.183012	1.47E-08	2.46E-08
AC008610	0.197255	1.030347	2.384993	9.02E-19	4.70E-18
AC112491	0.980106	1.973378	1.009658	0.001643	0.001939
AC008549	23.38095	11.31306	-1.04734	3.88E-14	1.11E-13
AC124016	0.256978	0.622836	1.27721	6.96E-07	1.04E-06
AC074117	0.472669	1.294549	1.453547	4.76E-21	4.64E-20
AC008771	1.045184	2.529414	1.275047	5.02E-19	2.88E-18
NOP14-AS	0.508031	1.086813	1.097115	4.54E-21	4.54E-20
GAS5	6.027653	33.72981	2.484356	3.75E-25	1.39E-23
LINC0170	0.124767	1.046643	3.068464	2.05E-20	1.57E-19
LINC0052	0.127875	0.637619	2.317958	2.50E-20	1.87E-19
DANCR	4.42438	11.78659	1.413599	4.06E-09	7.13E-09
AL355102.	0.350582	2.513658	2.841965	2.42E-05	3.28E-05
AL117336.	0.237875	0.826471	1.796759	5.49E-19	3.08E-18
ZNF529-A	0.294109	0.914214	1.63618	1.19E-20	9.40E-20

LINC01857	0.259608	0.534095	1.040763	0.000205	0.000261
VPS13B-D	0.732605	2.321344	1.663853	4.04E-15	1.29E-14
AC145207	0.195689	0.684148	1.805747	4.30E-19	2.53E-18
AL035071.	0.803633	1.998843	1.314556	6.45E-15	2.01E-14
AC048341	0.215025	1.087921	2.338998	3.03E-21	3.20E-20
AC026740	0.138208	1.35502	3.293399	2.29E-19	1.42E-18
AL451165.	2.105299	4.605082	1.129202	6.73E-12	1.54E-11
LINC01004	0.201823	0.842962	2.062378	2.09E-22	2.76E-21
AC084824	0.42867	1.024414	1.25686	1.66E-13	4.40E-13
AC027644	1.313399	2.823769	1.104317	8.82E-08	1.39E-07
LINC01093	30.82677	4.466404	-2.787	2.29E-25	1.06E-23
AC012146	0.325398	1.504259	2.208774	3.99E-20	2.90E-19
AL161669.	0.361577	1.433957	1.987625	5.16E-15	1.62E-14
PANK2-AS	0.23517	0.654008	1.475604	4.43E-13	1.14E-12
LINC02035	0.291287	0.618881	1.087221	1.71E-10	3.40E-10
SNHG15	0.907477	1.884032	1.05389	8.83E-06	1.23E-05
AC006042	0.156822	0.648316	2.047564	4.47E-09	7.80E-09
AC060780	0.228525	0.809819	1.82525	2.87E-12	6.79E-12
PARD3-AS	0.21016	0.590742	1.491039	1.97E-08	3.27E-08
AC010547	2.315825	1.131974	-1.03269	5.12E-24	1.18E-22
C2-AS1	0.245822	0.628038	1.353239	6.06E-08	9.70E-08
AC083843	0.297522	0.754353	1.342241	1.38E-08	2.31E-08
AC120053	0.55038	1.628419	1.564972	6.09E-20	4.29E-19
SNHG14	0.211369	0.637834	1.593418	0.006129	0.007054
AC004540	1.749296	0.567111	-1.62507	8.08E-20	5.53E-19
AL121899.	0.318121	2.390599	2.909724	1.11E-19	7.40E-19
LINC02163	0.00121	1.021286	9.721556	1.64E-14	4.91E-14
MYLK-AS1	0.097409	0.699674	2.844562	7.53E-27	9.34E-25
AC115618	2.291425	4.702419	1.037158	5.94E-11	1.27E-10
AP003352.	0.373765	1.541082	2.043742	2.65E-23	4.56E-22
DICER1-AS	0.353131	0.754735	1.095769	1.27E-11	2.85E-11
AC010326	1.238888	2.959211	1.256167	4.83E-17	1.96E-16
SNHG29	18.16456	37.66017	1.051913	5.89E-08	9.48E-08
AL391056.	0.022611	0.671633	4.892558	1.40E-07	2.16E-07
SREBF2-AS	0.190006	0.929223	2.289977	2.58E-26	2.12E-24
HSD11B1-	1.33292	3.547031	1.412022	0.000442	0.000541
AC009022	0.334902	0.709271	1.082599	1.28E-10	2.59E-10
HCG18	0.341767	0.924909	1.436299	2.01E-18	1.01E-17
AC005332	1.737848	4.635553	1.41544	2.31E-18	1.14E-17
SEMA3B-A	0.387422	1.398511	1.851916	2.63E-08	4.30E-08
LINC01836	0.229212	0.878819	1.938882	0.004285	0.00497
AL603839.	0.272785	0.734063	1.428142	4.54E-09	7.91E-09
MMP25-A	0.293517	0.729784	1.314025	3.40E-13	8.80E-13
ZSCAN16-	3.443201	7.500916	1.123316	1.64E-12	3.98E-12
AL133520.	0.545992	1.25653	1.202494	0.000172	0.000219
LINC01942	0.689695	1.450846	1.072865	2.74E-06	3.92E-06
FAM111A-	0.273121	0.698282	1.35427	7.48E-17	2.98E-16
NIFK-AS1	0.570199	1.264887	1.149471	3.89E-19	2.34E-18
TMEM44-A	2.565958	5.857021	1.19067	1.52E-12	3.73E-12
AL031058.	2.064092	4.936447	1.257966	3.36E-05	4.49E-05
SNHG8	19.47856	40.22252	1.046116	2.53E-11	5.47E-11
LINC01139	0.035456	0.881146	4.635298	0.044866	0.049259
LINC01124	0.949325	3.414409	1.846662	2.77E-05	3.72E-05
LINC00896	0.094482	0.652229	2.787265	7.72E-11	1.60E-10
AC022784	0.246774	1.013012	2.037387	5.16E-07	7.76E-07
AC016747	1.090886	2.809532	1.36483	8.60E-27	9.34E-25
OTUD6B-A	1.62141	3.280372	1.016611	3.27E-18	1.57E-17
HAGLR	0.011622	1.507579	7.01919	1.39E-27	3.43E-25

LINC01484	0.203283	0.553471	1.445023	1.46E-09	2.65E-09
AL355353.	1.183044	2.465145	1.059169	0.000336	0.000418
AL359513.	0.120214	0.575208	2.258478	4.98E-17	2.02E-16
AL354836.	0.4877	1.49624	1.617275	2.75E-15	8.89E-15
AL441992.	1.048315	3.2731	1.642585	1.85E-20	1.43E-19
AC068506	0.12093	0.753633	2.639685	0.000148	0.000189
SNHG20	0.361795	1.288704	1.832675	3.89E-24	9.83E-23
LINC00886	0.43178	1.150869	1.414354	4.27E-05	5.68E-05
FAM99B	3.77454	1.807266	-1.06249	1.03E-14	3.16E-14
AL360181.	1.310395	2.764978	1.077266	0.027017	0.029929
AC008735	0.370896	1.546773	2.060174	6.96E-16	2.47E-15
AL365181.	0.044872	0.872083	4.28057	2.53E-16	9.56E-16
LENG8-AS	0.190327	0.882615	2.213303	1.87E-24	5.13E-23
AC020915	0.384038	1.193137	1.63544	1.47E-22	1.98E-21
AC009065	0.169893	0.610909	1.846331	5.94E-21	5.43E-20
AC016773	0.060701	0.578569	3.252689	3.47E-25	1.39E-23
ZNF687-A	0.677052	1.868192	1.464304	4.37E-15	1.39E-14
AC092171	0.320882	3.082692	3.264074	1.15E-27	3.43E-25
AL121832.	0.779115	2.727053	1.807435	1.49E-19	9.83E-19
AC024075	0.174663	0.549903	1.654606	5.20E-10	9.98E-10
AC012510	0.287811	0.949093	1.721429	8.56E-17	3.37E-16
LINC00942	0.00615	1.555603	7.982678	1.01E-08	1.73E-08
AC099508	1.932979	0.547204	-1.82067	4.34E-19	2.53E-18
AP000593.	0.02346	1.496655	5.995414	1.27E-10	2.58E-10
LINC00685	0.125699	0.870312	2.791565	3.60E-23	5.79E-22
AC012313	0.864041	1.757568	1.024408	9.24E-07	1.38E-06
LINC00221	0.002712	1.10443	8.669661	1.59E-07	2.44E-07
IDH1-AS1	0.466803	1.204681	1.367767	1.70E-09	3.08E-09
AP000759.	0.605085	1.53736	1.345246	3.18E-14	9.23E-14
LINC02041	0.080913	1.092878	3.755614	0.002729	0.00319
AC015849	0.184326	0.56545	1.617139	7.65E-10	1.45E-09
MALAT1	2.157594	6.152177	1.511673	1.04E-15	3.56E-15
AC111000	0.122345	1.127003	3.20347	1.78E-17	7.60E-17
AC068473	0.419292	1.157338	1.464782	1.80E-18	9.12E-18
AL391244.	0.351646	1.049788	1.577903	1.31E-20	1.02E-19
AC084824	0.330474	0.850634	1.364002	1.71E-15	5.58E-15
DNAJC3-D	0.431365	1.022508	1.24513	7.99E-11	1.65E-10
EIF3J-DT	0.46915	1.015648	1.11428	7.32E-16	2.55E-15
AC009686	0.171282	0.670775	1.969452	7.12E-11	1.49E-10
AC079305	0.147634	0.58304	1.981569	1.24E-07	1.92E-07
AC004816	0.145398	0.996639	2.777066	1.32E-22	1.80E-21
LINC00342	0.389269	1.131618	1.539549	2.17E-14	6.41E-14
AC023906	0.10081	0.870858	3.110793	0.000286	0.000361
LINC00623	0.739846	1.692711	1.194039	2.17E-11	4.72E-11
AC010883	0.422618	1.25078	1.5654	6.47E-13	1.63E-12
AL356234.	0.05398	0.826058	3.93575	7.62E-14	2.08E-13
AC132872	0.521036	1.942457	1.898427	1.87E-23	3.46E-22
AC007773	0.13765	0.707799	2.362333	4.93E-18	2.31E-17
LINC02241	0.000426	0.888621	11.02666	1.28E-12	3.15E-12
LINC01480	0.049733	0.881198	4.147179	3.55E-08	5.80E-08
AC005332	0.359772	1.790952	2.31557	1.06E-26	9.84E-25
AL118558.	0.591829	1.33673	1.175456	1.64E-12	3.98E-12
CD2BP2-D	1.001605	2.272652	1.182063	7.14E-12	1.63E-11
AC005920	0.45807	1.228889	1.423713	0.000375	0.000465
LNCTAM3-	0.319557	0.918125	1.522618	9.60E-15	2.96E-14
AL161668.	6.377865	2.54853	-1.32341	4.74E-16	1.73E-15
LINC02062	0.20721	0.548325	1.403935	1.58E-11	3.52E-11
AC095057	0.171051	0.559146	1.708795	2.95E-12	6.95E-12

AC011700	0.23329	0.548133	1.232399	3.56E-06	5.03E-06
AC023090	0.007746	0.626512	6.337672	7.54E-09	1.31E-08
ELF3-AS1	0.433825	2.177377	2.327406	1.04E-19	6.98E-19
AL355574.	0.219373	0.91761	2.064496	6.62E-19	3.63E-18
AC107375	0.162585	0.732007	2.170663	9.91E-26	5.24E-24
AC010761	0.219843	0.749769	1.76997	8.80E-18	3.97E-17
AC011468	0.290868	1.169254	2.007151	8.44E-19	4.46E-18
AL365181.	0.226268	4.241738	4.22855	7.58E-18	3.44E-17
ASH1L-AS	0.294663	0.774384	1.393984	5.26E-18	2.45E-17
AP000254.	1.254763	2.521725	1.006996	6.47E-13	1.63E-12
AL031186.	0.123074	0.595767	2.275221	4.87E-21	4.68E-20
AC068580	0.204722	0.892079	2.123503	2.97E-13	7.73E-13
AC034231	0.254385	0.768437	1.594914	2.31E-15	7.51E-15
MAFG-DT	0.119233	1.476454	3.630285	7.98E-25	2.57E-23
AC002398	0.309954	0.894128	1.528426	4.96E-15	1.57E-14
MIR4435-2	0.372212	2.014476	2.436208	9.95E-23	1.42E-21
PSORS1C3	0.144938	0.911937	2.653502	1.35E-09	2.48E-09
AC013275	0.098836	0.983149	3.3143	1.05E-07	1.64E-07
AC068888	0.742697	1.509609	1.023329	1.96E-10	3.89E-10
ELFN1-AS	0.04566	0.910602	4.317824	0.005869	0.006775
ARHGAP2	0.177379	0.584585	1.720578	8.63E-21	7.17E-20
AC022424	0.008662	0.788276	6.507832	1.47E-07	2.27E-07
AC015813	0.209155	0.796465	1.929041	6.08E-17	2.44E-16
LINC0154	0.128305	2.128594	4.052255	0.00056	0.000681
AC025265	0.154723	0.565949	1.870984	4.97E-12	1.15E-11
RNASEH1-	1.306506	2.769461	1.083891	9.79E-16	3.38E-15
AC019117	0.246056	0.899713	1.870478	0.000429	0.000527
PTOV1-AS	0.473182	1.231985	1.380518	4.84E-23	7.62E-22
LINC0084	2.098642	4.264081	1.022779	3.39E-16	1.26E-15
AC004148	0.262708	0.790841	1.589929	1.07E-15	3.65E-15
AL359504.	0.175786	0.544795	1.631892	6.39E-14	1.76E-13
AC018904	0.950887	2.41173	1.342723	4.94E-16	1.79E-15
AC007405	0.903436	2.434416	1.430082	8.72E-15	2.70E-14
AL035461.	0.370123	2.059544	2.476249	6.52E-20	4.55E-19
AC142472	0.218401	0.663329	1.602746	5.37E-19	3.03E-18
AC020978	4.697383	2.171596	-1.1131	1.64E-15	5.38E-15
AC040977	1.058995	3.028163	1.515747	3.00E-18	1.45E-17
AL645933.	0.712656	1.56922	1.13877	1.98E-08	3.29E-08
RUSC1-AS	0.286733	1.654775	2.528857	1.14E-24	3.38E-23
LINC0260	0.686024	1.567989	1.192585	3.43E-13	8.85E-13
AC087741	0.352441	1.30265	1.885998	1.72E-13	4.54E-13
AC009005	0.189461	1.413915	2.899726	3.69E-20	2.70E-19
AC015871	0.359205	0.808541	1.170513	5.86E-13	1.49E-12
LINC0095	0.319173	0.731721	1.196957	3.95E-08	6.42E-08
AFDN-DT	0.31699	0.691875	1.126076	1.38E-09	2.53E-09
AL135999.	0.165959	0.647764	1.964643	1.89E-17	7.92E-17
TMPO-AS	0.223928	1.154242	2.365841	1.34E-23	2.75E-22
AC016394	0.189738	0.83029	2.129609	8.24E-21	7.09E-20
TAF1A-AS	0.747174	1.499026	1.004508	1.23E-09	2.28E-09
AC024075	0.80442	2.408968	1.582394	4.93E-21	4.68E-20
AC009275	0.087308	0.733148	3.069928	1.02E-08	1.74E-08
LINC0023	0.10789	0.636681	2.561005	1.43E-05	1.96E-05
KCNMB2-.	0.017109	0.749221	5.452522	1.26E-14	3.84E-14
BX284668.	3.892017	8.167327	1.069346	1.26E-08	2.14E-08
AC011477	0.542014	1.39663	1.365547	2.86E-09	5.10E-09
AC078993	0.026049	0.834589	5.001779	3.55E-10	6.90E-10
NEAT1	5.481795	12.6457	1.205927	9.87E-09	1.70E-08
SCAT8	0.273798	1.038534	1.923365	3.09E-05	4.15E-05

AP003174.	0.086242	0.559118	2.69669	1.90E-11	4.19E-11
AL365330.	0.376062	0.925295	1.298943	1.51E-11	3.39E-11
AC007541	0.39944	1.043334	1.385149	1.04E-15	3.56E-15
AC020765	0.257708	0.923857	1.841933	4.11E-19	2.44E-18
LINC0066!	0.119604	1.405468	3.554708	1.62E-10	3.23E-10
LINC0153	0.326395	0.746582	1.193683	2.13E-12	5.12E-12
LINC0176	17.83771	8.809328	-1.01783	2.66E-12	6.34E-12
AC016888	2.416702	6.058208	1.325851	2.70E-12	6.41E-12
HCP5	3.080273	8.09691	1.394313	1.13E-09	2.11E-09
AL031985.	0.218939	0.7233	1.724069	8.18E-20	5.55E-19
AL606489.	0.055938	0.834341	3.898725	8.23E-24	1.84E-22
SNHG3	0.633349	3.314609	2.387767	3.52E-23	5.78E-22
HEIH	5.341754	12.86642	1.268225	2.07E-24	5.47E-23
LMNTD2-/	0.195997	0.824573	2.072817	7.63E-14	2.08E-13
AC068580	0.311359	1.017235	1.708003	3.09E-14	9.04E-14
PRRT3-AS	0.442701	1.748381	1.981615	2.87E-10	5.64E-10
AC005332	0.232729	0.563755	1.276415	1.78E-11	3.94E-11
AP002360.	1.214584	4.067104	1.743539	8.08E-19	4.33E-18
SNHG10	0.455102	1.518934	1.738798	1.56E-23	2.99E-22
AC087741	0.18484	0.716305	1.954298	6.13E-19	3.41E-18
U62317.2	1.545934	3.76831	1.285439	5.74E-16	2.06E-15
NRAV	0.820404	2.090722	1.349595	3.98E-19	2.37E-18
MAGI2-AS	1.564102	0.758725	-1.04369	1.16E-13	3.10E-13
LINC0282	0.01707	0.838233	5.617827	7.33E-05	9.62E-05
SLC6A1-A	0.151585	0.554465	1.87097	4.88E-06	6.87E-06
AC139530	0.504627	1.532106	1.602227	9.25E-21	7.52E-20
AC106820	0.182074	0.936768	2.363168	6.44E-19	3.56E-18
VPS9D1-A	0.293666	0.823905	1.488303	1.16E-06	1.71E-06
AP001372.	0.554063	1.199578	1.114406	1.22E-15	4.10E-15
AL355488.	0.159005	0.990814	2.639539	9.07E-25	2.80E-23
AC055822	0.22162	0.704284	1.66807	4.86E-14	1.37E-13
SNHG1	1.243861	6.041754	2.280142	4.47E-26	3.01E-24
AC040970	0.106725	0.629852	2.561114	7.47E-06	1.04E-05
ZNF793-A	0.1002	0.571155	2.510992	0.000598	0.000725
MELTF-AS	0.112246	0.961025	3.097909	7.08E-21	6.24E-20
AP000894.	0.374369	1.259622	1.75046	3.26E-13	8.48E-13
SNHG7	1.412275	6.609583	2.226538	1.87E-24	5.13E-23
CRIM1-DT	0.851886	3.148073	1.885737	0.000112	0.000144
WNT5A-A	0.191036	0.608341	1.671033	0.003879	0.004507
AL392172.	1.84866	5.774383	1.643187	2.30E-21	2.50E-20
SNHG12	0.473602	1.811267	1.935252	7.10E-22	8.62E-21
AC104794	1.431963	3.121834	1.124399	4.55E-14	1.29E-13
AC027796	0.106294	0.583389	2.456399	1.25E-17	5.51E-17
ZNF503-A	0.411787	1.040383	1.337146	1.67E-17	7.18E-17
AC090587	0.486699	1.009292	1.052243	4.96E-08	8.01E-08
PTOV1-AS	0.453243	1.55688	1.780301	2.66E-18	1.30E-17
MHENCR	1.64299	4.408032	1.42381	4.83E-17	1.96E-16
AC125257	1.260418	2.688782	1.093051	1.38E-21	1.53E-20
AL353622.	0.247943	0.804718	1.698475	1.13E-14	3.46E-14
LINC0051	0.157961	0.647675	2.035702	5.61E-07	8.38E-07
BAIAP2-D	1.719264	4.877977	1.504492	8.92E-19	4.68E-18
AC092171	0.415698	1.573045	1.919952	1.35E-17	5.91E-17
APTR	1.110512	2.650322	1.254943	1.32E-22	1.80E-21
GPRC5D-/	0.401211	0.934751	1.220223	1.66E-19	1.07E-18
PRANCR	0.415161	1.046368	1.333649	9.09E-18	4.08E-17
AC023157	0.545236	1.186093	1.121264	1.01E-09	1.90E-09
LINC0202	5.577541	1.867271	-1.5787	1.67E-19	1.07E-18
AC000123	0.433409	0.95974	1.146914	1.65E-12	4.00E-12

AC005586	0.251212	0.582299	1.212852	1.09E-06	1.62E-06
ZNF213-A	0.499615	1.267142	1.342689	9.14E-21	7.52E-20
AC009237	0.246232	0.665483	1.434382	5.71E-12	1.31E-11
U62317.1	0.259178	0.800768	1.627444	1.35E-08	2.27E-08
FOXD2-AS	0.225472	1.579585	2.808525	5.05E-21	4.73E-20
C2CD4D-/	1.028418	4.638335	2.17318	5.25E-19	2.99E-18
AC131009	0.4517	1.221894	1.435683	1.75E-12	4.23E-12
AC026462	1.320374	3.73676	1.500841	5.91E-05	7.79E-05
AC068987	0.268475	1.605063	2.57977	5.60E-14	1.57E-13
AC090589	0.359482	0.774625	1.107579	5.67E-08	9.15E-08
YTHDF3-A	0.868331	2.105716	1.277994	1.34E-10	2.70E-10
AP003486.	0.341558	0.827403	1.276461	1.61E-15	5.33E-15
AC080129	0.121195	0.789454	2.703525	1.79E-17	7.61E-17
AJ009632.	0.319346	1.330368	2.058634	0.020947	0.023521
AC074212	0.153294	0.633311	2.046613	7.80E-23	1.15E-21
AP002807.	0.172393	0.861442	2.321051	3.65E-18	1.74E-17
AC132192	0.117529	0.973848	3.050686	4.78E-28	3.43E-25
NRSN2-AS	0.361432	0.952883	1.398574	4.25E-12	9.90E-12
LINC0178(	0.300857	0.910318	1.597294	8.38E-14	2.26E-13
AC012313	0.609874	1.304765	1.097206	9.70E-17	3.78E-16
GAPLINC	0.071529	0.893981	3.643653	4.51E-14	1.28E-13
LINC01671	0.162633	0.682322	2.068836	0.021908	0.024526
AL117379.	0.126153	0.619514	2.295958	1.10E-21	1.24E-20
BACE1-AS	0.560946	2.149493	1.938062	5.04E-25	1.78E-23
LUCAT1	0.021123	0.578824	4.776251	1.12E-08	1.91E-08
RAD51-AS	0.343281	1.056517	1.621855	7.47E-20	5.16E-19
AC006026	0.2707	0.648452	1.260303	0.001284	0.001528
AC090164	0.076786	0.727029	3.243102	7.20E-14	1.98E-13
AL844908.	0.221688	0.550804	1.313007	1.09E-07	1.71E-07
AC079466	0.038081	4.695623	6.946087	1.47E-06	2.17E-06
CR936218.	0.312461	0.861487	1.463154	0.001408	0.001673
AC007406	0.16021	1.189949	2.892865	1.98E-06	2.85E-06
TMEM147-	0.183058	0.685395	1.904638	3.38E-22	4.31E-21
CEBPA-DT	1.43906	4.884097	1.762965	3.55E-15	1.14E-14
AC021078	0.380296	0.829084	1.124397	9.11E-14	2.45E-13
AL162595.	0.249471	0.77042	1.626774	1.36E-17	5.93E-17
AC005229	0.660627	1.681631	1.347953	1.86E-19	1.19E-18
AC027307	2.892197	8.621631	1.575795	4.19E-24	1.00E-22
MID1IP1-/	0.322096	1.010146	1.649002	2.55E-09	4.56E-09
AC008608	0.970355	2.129741	1.134093	1.73E-09	3.12E-09
AC104534	0.124183	0.870038	2.80861	5.88E-11	1.26E-10
AL139287.	0.856955	2.656586	1.632282	2.35E-21	2.52E-20
AC010973	0.110305	0.626642	2.506145	7.06E-26	4.35E-24
AC022144	0.523767	1.218033	1.217555	0.000648	0.000785
KMT2E-AS	2.387686	5.900016	1.305106	1.52E-13	4.04E-13
AP006621.	0.24696	1.609354	2.704131	1.54E-16	5.85E-16
AC005261	1.477345	2.973788	1.009295	9.39E-18	4.16E-17
ZEB1-AS1	0.274888	0.805408	1.550878	3.63E-22	4.56E-21
RAB30-DT	1.130121	2.478681	1.133095	2.14E-18	1.07E-17
AC007038	0.177519	0.604213	1.767087	3.11E-19	1.90E-18
AC005261	1.635385	3.93295	1.265981	2.65E-23	4.56E-22
MIR4458H	0.66621	1.560526	1.227984	1.95E-09	3.50E-09
AC006449	0.636535	1.282471	1.010615	1.33E-09	2.45E-09
AL390728.	0.99226	3.999798	2.011137	1.78E-18	9.08E-18
AL591895.	2.803406	10.15937	1.857558	3.09E-21	3.22E-20
LINC0143(	0.088615	1.690845	4.254042	5.70E-06	7.98E-06
MUC20-O	0.327767	0.84424	1.364981	5.80E-18	2.66E-17
LINC00997	0.581848	1.178157	1.017817	8.01E-16	2.78E-15

STPG3-AS	0.139222	0.77431	2.475524	2.17E-14	6.41E-14
AC145343	0.145612	0.786465	2.433257	5.73E-18	2.65E-17
AC004918	0.394433	1.168577	1.5669	4.77E-18	2.25E-17
AC108134	0.14337	0.57769	2.010556	7.32E-14	2.00E-13
AC253536	0.418573	1.685762	2.00985	3.37E-09	5.96E-09
LINC0118(	0.076483	0.778008	3.346572	1.61E-14	4.83E-14
AC024060	0.86913	2.733276	1.652988	8.19E-23	1.19E-21
SNHG17	1.42856	3.703286	1.374244	3.19E-16	1.19E-15
SBF2-AS1	0.233662	0.755556	1.693116	3.38E-22	4.31E-21
SNHG21	0.193549	0.642201	1.730323	6.92E-21	6.17E-20
AC079922	0.431565	0.885701	1.037243	4.72E-13	1.20E-12
LINC0242ε	10.75337	4.042471	-1.41148	4.04E-17	1.67E-16
ALDH1L1-	0.319396	0.689547	1.110303	0.003118	0.003628
AC007996	0.225625	0.713521	1.661032	1.58E-15	5.24E-15
Z98257.1	0.170202	0.806715	2.244809	1.08E-08	1.84E-08
AC243964	0.765912	1.811367	1.241828	1.96E-06	2.84E-06
LINC0108(	0.248189	1.233953	2.313775	3.82E-26	2.83E-24
AC008915	0.92107	1.967004	1.094618	1.56E-15	5.22E-15
AL360181.	0.334574	0.846639	1.33942	5.23E-10	9.99E-10
AC139100	0.144009	0.980064	2.766721	6.93E-22	8.55E-21
SNHG32	9.754471	36.20809	1.892177	3.75E-25	1.39E-23
DSCR8	0.002448	1.188885	8.924002	8.02E-09	1.39E-08
AL021707.	0.447574	1.213484	1.438958	5.47E-12	1.26E-11
AL022328.	0.734639	2.099015	1.514605	6.21E-17	2.48E-16
AC026401	0.913817	3.270511	1.839539	2.24E-18	1.11E-17
SNHG9	3.260921	9.528414	1.546957	1.33E-10	2.69E-10
LINC0117(	0.43653	1.210682	1.471667	1.29E-15	4.34E-15
