# Supplementary material for: Construction of a novel immune-related lncRNA signature and its potential to predict the immune status of patients with hepatocellular carcinoma
Source: BMC Cancer. 2021 Dec 19;21:1347. doi: 10.1186/s12885-021-09059-x (PMC8684648; doi:10.1186/s12885-021-09059-x)
Supplement: Supplementary file 3 — Additional file 3. [file 12885_2021_9059_MOESM3_ESM.pdf]

Id	futime	fustat	age	gender	grade	stage	T	M
TCGA-DD-	2415	0	68	MALE	G2	Stage II	T2	M0
TCGA-KR-	65	1	65	MALE	G1	Stage I	T1	M0
TCGA-DD-	2456	1	61	FEMALE	G2	Stage I	T1	M0
TCGA-CC-	347	1	63	MALE	G3	Stage IIIA	T3	M0
TCGA-G3-	361	0	38	MALE	G2	Stage II	T2	M0
TCGA-DD-	763	0	51	MALE	G3	Stage I	T1	M0
TCGA-DD-	555	0	45	FEMALE	G4	Stage IIIA	T3a	M0
TCGA-CC-	300	1	45	MALE	G3	Stage IIIA	T3	M0
TCGA-DD-	612	1	37	MALE	G3	Stage IIIA	T3	M0
TCGA-DD-	1242	0	51	MALE	G4	Stage I	T1	M0
TCGA-G3-	860	0	68	MALE	G2	Stage I	T1	M0
TCGA-DD-	644	0	72	MALE	G2	Stage I	T1	M0
TCGA-LG-	425	0	48	MALE	G2	Stage I	T1	M0
TCGA-DD-	784	0	73	MALE	G2	Stage I	T1	M0
TCGA-DD-	1989	0	55	MALE	G2	Stage IIIA	T3	M0
TCGA-2Y-	697	0	70	MALE	G2	Stage I	T1	MX
TCGA-5C-	20	0	61	MALE	G1	Stage II	T2	M0
TCGA-EP-	357	0	52	FEMALE	G3	Stage IIIA	T3a	MX
TCGA-DD-	283	1	72	FEMALE	G2	Stage I	T1	M0
TCGA-UB-	52	1	67	MALE	G3	Stage I	T1	MX
TCGA-DD-	2425	0	55	FEMALE	G3	Stage II	T2	M0
TCGA-5C-	322	0	70	MALE	G2	Stage I	T1	M0
TCGA-ED-	482	0	60	FEMALE	G2	Stage II	T2	M0
TCGA-BC-	837	1	69	MALE	G2	unknow	T2	MX
TCGA-DD-	643	1	77	MALE	G3	Stage II	T2	M0
TCGA-ED-	423	0	74	MALE	G2	Stage I	T1	M0
TCGA-G3-	372	0	50	MALE	G1	Stage I	T1	M0
TCGA-ZS-	1386	1	73	MALE	G2	Stage II	T2	MX
TCGA-GJ-	67	1	68	MALE	G2	Stage I	T1	MX
TCGA-G3-	27	1	83	FEMALE	G1	Stage I	T1	M0
TCGA-DD-	458	0	45	MALE	G3	Stage I	T1	M0
TCGA-DD-	1711	0	46	MALE	G1	Stage I	T1	M0
TCGA-CC-	97	1	44	MALE	G2	Stage II	T2	M0
TCGA-UB-	500	0	59	MALE	G3	Stage IIIA	T3a	MX
TCGA-CC-	649	1	59	MALE	G1	Stage IIIA	T3	M0
TCGA-4R-	262	1	66	MALE	G2	Stage II	T2	MX
TCGA-DD-	701	0	65	FEMALE	G1	Stage IIIA	T3a	M0
TCGA-CC-	250	0	60	FEMALE	G2	Stage IIIC	T4	M0
TCGA-2Y-	1271	1	64	MALE	G2	Stage I	T1	MX
TCGA-BC-	1423	1	81	MALE	G1	unknow	T3	MX
TCGA-UB-	486	0	51	MALE	G2	Stage I	T1	MX
TCGA-ED-	6	0	54	MALE	G2	Stage IIIA	T3a	M0
TCGA-XR-	693	1	74	MALE	G1	Stage I	T1	MX
TCGA-CC-	399	0	54	MALE	G3	Stage IIIA	T3	M0
TCGA-DD-	436	0	59	MALE	G3	Stage II	T2	M0
TCGA-K7-	512	0	66	MALE	G2	Stage I	T1	MX
TCGA-CC-	102	1	71	MALE	G2	Stage IIIA	T3	M0
TCGA-DD-	171	1	67	MALE	G3	Stage I	T1	M0
TCGA-ED-	56	1	50	FEMALE	G3	Stage IIIA	T3a	M0
TCGA-DD-	2759	1	70	MALE	G2	Stage I	T1	M0
TCGA-DD-	602	0	20	FEMALE	G3	Stage I	T1	M0
TCGA-DD-	2542	1	53	MALE	G2	Stage IIIA	T3	M0
TCGA-HP-	91	1	78	MALE	G2	Stage I	T1	M0
TCGA-DD-	558	1	64	FEMALE	G2	Stage IVB	T4	M1
TCGA-DD-	638	0	51	MALE	G3	Stage I	T1	M0
TCGA-CC-	272	1	48	MALE	G2	Stage IIIB	T4	M0
TCGA-NI-	606	0	74	MALE	G3	Stage I	T1	MX

TCGA-BW	20	0	50	MALE	G2	Stage IIIA	T3a	MX
TCGA-CC-	304	1	45	MALE	G2	Stage IIIA	T3	M0
TCGA-DD	394	1	57	FEMALE	G3	Stage I	T1	M0
TCGA-GJ-	879	0	59	MALE	G2	Stage I	T1	MX
TCGA-DD	2202	0	46	MALE	G2	Stage II	T2	M0
TCGA-G3-	671	0	65	MALE	G2	Stage IIIB	T3b	M0
TCGA-CC-	0	0	52	FEMALE	G2	Stage IIIA	T3a	M0
TCGA-DD	1295	0	43	MALE	G2	Stage I	T1	M0
TCGA-2Y-	1939	0	55	FEMALE	G2	Stage I	T1	MX
TCGA-MI-	698	0	63	MALE	G2	Stage II	T2	M0
TCGA-FV-	2486	1	78	MALE	G2	Stage IIIA	T3	M0
TCGA-PD-	639	1	58	FEMALE	G2	Stage IIIB	T4	M0
TCGA-DD	931	1	64	FEMALE	G2	Stage IVB	T4	M1
TCGA-DD	2301	0	65	MALE	G3	Stage I	T1	M0
TCGA-DD	1855	0	69	MALE	G2	Stage I	T1	M0
TCGA-DD	1231	0	51	MALE	G4	Stage I	T1	M0
TCGA-XR-	898	0	58	MALE	G2	Stage I	T1	M0
TCGA-UB-	314	0	69	FEMALE	G1	Stage I	unknow	MX
TCGA-CC-	217	1	57	MALE	G2	Stage IIIA	T3	M0
TCGA-BC-	387	0	69	MALE	G1	Stage I	T1	M0
TCGA-DD	566	0	50	MALE	G2	Stage I	T1	M0
TCGA-CC-	101	1	50	MALE	G3	Stage IIIA	T3	M0
TCGA-ZP-	21	0	56	FEMALE	G2	unknow	T1	MX
TCGA-EP-	303	0	76	MALE	G2	Stage I	T1	MX
TCGA-CC-	278	1	61	MALE	G1	Stage IIIA	T3	M0
TCGA-CC-	129	1	35	MALE	G1	Stage IIIA	T3	M0
TCGA-G3-	1779	0	73	MALE	G3	Stage II	T2	M0
TCGA-WÇ	30	0	71	FEMALE	G3	unknow	T3a	M0
TCGA-G3-	655	0	58	MALE	G2	Stage I	T1	M0
TCGA-DD	170	0	66	MALE	G3	Stage II	T2	M0
TCGA-DD	141	0	59	FEMALE	G2	Stage I	T1	M0
TCGA-UB-	327	0	60	MALE	G2	Stage II	T2	MX
TCGA-RG-	1098	0	69	MALE	G2	Stage II	T2	M0
TCGA-MI-	630	0	61	MALE	G1	unknow	T2	MX
TCGA-DD	1718	0	38	MALE	G2	Stage II	T2	M0
TCGA-EP-	19	1	62	MALE	G3	Stage I	T1	MX
TCGA-DD	898	0	59	MALE	G4	Stage I	T1	MX
TCGA-DD	2018	0	61	MALE	G1	Stage IIIA	T3	M0
TCGA-T1-	23	0	68	MALE	G2	unknow	T1	M0
TCGA-DD	1210	1	80	FEMALE	G2	Stage IIIA	T3	M0
TCGA-CC-	382	0	56	MALE	G3	Stage II	T2	M0
TCGA-DD	2028	0	58	MALE	G3	Stage I	T1	M0
TCGA-DD	690	0	65	FEMALE	G3	Stage IIIB	T3b	M0
TCGA-BD-	1363	0	69	MALE	G2	unknow	T2	MX
TCGA-G3-	673	0	80	MALE	G2	Stage I	T1	M0
TCGA-RC-	0	0	24	MALE	G3	Stage II	T2	M0
TCGA-DD	2324	0	74	FEMALE	G3	Stage I	T1	M0
TCGA-BC-	711	1	76	MALE	G3	unknow	T4	MX
TCGA-CC-	248	0	68	MALE	G2	Stage IIIA	T3	M0
TCGA-DD	415	1	23	MALE	G3	Stage II	T2	M0
TCGA-DD	810	0	55	MALE	G4	Stage I	T1	M0
TCGA-BC-	91	1	50	MALE	G3	unknow	T4	MX
TCGA-CC-	344	1	39	FEMALE	G3	Stage IIIA	T3	M0
TCGA-RC-	472	0	59	MALE	G3	Stage I	T1	M0
TCGA-CC-	129	1	48	MALE	G2	Stage II	T2	M0
TCGA-UB-	214	1	56	MALE	G2	Stage IIIA	T3a	MX
TCGA-DD	107	1	66	FEMALE	G3	Stage I	T1	M0
TCGA-BC-	837	1	76	MALE	G1	unknow	T4	MX

TCGA-DD	2184	0	62	MALE	G3	Stage I	T1	M0
TCGA-BC-	2116	1	51	FEMALE	G1	unknow	T1	MX
TCGA-ED-	390	0	20	FEMALE	G3	Stage II	T2	M0
TCGA-DD	9	1	85	FEMALE	G3	Stage I	T1	M0
TCGA-BC-	562	0	66	FEMALE	G3	Stage IIIC	T4	M0
TCGA-DD	554	0	60	MALE	G3	Stage II	T2	M0
TCGA-BC-	34	1	62	FEMALE	G2	Stage I	T1	MX
TCGA-DD	9	0	70	MALE	G2	Stage I	T1	M0
TCGA-DD	1067	0	51	MALE	G3	Stage II	T2	M0
TCGA-DD	1633	0	69	MALE	G2	Stage I	T1	M0
TCGA-2Y-	848	1	82	FEMALE	G2	Stage II	T2	MX
TCGA-DD	1085	0	77	FEMALE	G1	Stage I	T1	M0
TCGA-DD	1804	0	39	MALE	G3	Stage I	T1	M0
TCGA-ED-	296	1	51	FEMALE	G3	Stage IIIA	T3a	M0
TCGA-G3-	594	0	52	MALE	G2	Stage II	T2	M0
TCGA-2Y-	1731	0	64	FEMALE	G3	Stage I	T1	MX
TCGA-ED-	6	0	61	MALE	G2	Stage II	T2	M0
TCGA-DD	1618	0	69	MALE	G3	Stage II	T2	M0
TCGA-DD	1622	1	68	MALE	G3	Stage IIIA	T3	M0
TCGA-DD	719	0	59	FEMALE	G3	Stage I	T1	M0
TCGA-DD	1149	1	42	MALE	G3	Stage II	T2	M0
TCGA-ZS-	2412	0	64	MALE	G2	Stage II	T2	MX
TCGA-RC-	9	0	75	MALE	G3	Stage II	T2	M0
TCGA-5R-	364	0	57	MALE	G2	Stage II	T2	M0
TCGA-CC-	103	1	67	MALE	G1	Stage IIIC	T4	M0
TCGA-DD	1876	0	40	MALE	G3	Stage I	T1	M0
TCGA-ZP-	782	0	66	FEMALE	G1	unknow	T1	MX
TCGA-ZP-	395	0	64	FEMALE	G1	unknow	T1	MX
TCGA-ZS-	1241	0	79	FEMALE	G1	Stage II	T2	MX
TCGA-DD	1823	0	56	MALE	G2	Stage I	T1	M0
TCGA-XR-	1339	0	43	FEMALE	G2	Stage I	T1	MX
TCGA-DD	827	1	29	FEMALE	G2	Stage III	T3	M0
TCGA-BC-	444	0	64	MALE	G3	Stage II	T2	M0
TCGA-DD	1008	0	67	FEMALE	G3	Stage IIIC	T2	M0
TCGA-MI-	507	0	61	MALE	G2	Stage IIIC	T4	M0
TCGA-DD	1900	0	38	MALE	G2	Stage I	T1	M0
TCGA-G3-	621	0	70	MALE	G2	Stage II	T2	M0
TCGA-ED-	400	0	53	FEMALE	G3	Stage II	T2	M0
TCGA-EP-	334	0	46	FEMALE	G2	Stage I	T1	MX
TCGA-DD	561	0	67	MALE	G2	Stage II	T2	M0
TCGA-CC-	0	0	57	MALE	G2	Stage IIIA	T3	M0
TCGA-WQ	395	0	62	MALE	G2	Stage II	T2	M0
TCGA-G3-	359	1	51	MALE	G3	Stage IIIC	T4	M0
TCGA-CC-	363	0	54	MALE	G2	Stage IIIA	T3	M0
TCGA-G3-	480	0	48	MALE	G2	Stage I	T1	M0
TCGA-DD	1769	0	48	MALE	G3	Stage II	T2	M0
TCGA-DD	1560	1	57	FEMALE	G2	Stage I	T1	M0
TCGA-DD	365	1	68	MALE	G3	Stage I	T1	M0
TCGA-BD-	409	0	75	FEMALE	G2	Stage I	T1	M0
TCGA-ED-	386	0	58	MALE	G3	Stage II	T2	M0
TCGA-DD	79	0	67	MALE	G3	Stage I	T1	M0
TCGA-QA	94	0	48	MALE	G2	Stage II	T2	MX
TCGA-DD	636	0	58	MALE	G4	Stage I	T1	M0
TCGA-2Y-	357	0	68	FEMALE	G2	Stage I	T1	MX
TCGA-2Y-	757	1	64	FEMALE	G3	Stage II	T2	MX
TCGA-DD	44	0	66	MALE	G1	Stage I	T1	M0
TCGA-RC-	579	0	66	MALE	G2	Stage I	T1	M0
TCGA-BC-	498	0	68	FEMALE	G3	Stage II	T2	M0

TCGA-DD	2752	0	48	MALE	G1	Stage I	T1	M0
TCGA-ED-	408	0	47	MALE	G2	Stage II	T2	M0
TCGA-DD	1424	0	43	MALE	G3	Stage I	T1	M0
TCGA-3K-	396	0	65	MALE	G1	Stage IIIB	T3b	MX
TCGA-G3-	447	0	76	MALE	G2	Stage I	T1	MX
TCGA-FV-	194	1	75	MALE	unknow	Stage I	T1	MX
TCGA-BD-	1115	0	62	MALE	G2	Stage II	T2	MX
TCGA-DD	555	0	55	FEMALE	G2	Stage I	T1	M0
TCGA-DD	1450	0	61	MALE	G3	Stage I	T1	M0
TCGA-DD	183	0	46	MALE	G2	Stage I	T1	M0
TCGA-2Y-	260	0	66	MALE	G2	Stage I	T1	MX
TCGA-CC-	219	0	24	FEMALE	G1	Stage IIIA	T3	M0
TCGA-XR-	925	0	16	MALE	G1	Stage IIIA	T3	MX
TCGA-CC-	140	1	74	MALE	G2	Stage IIIA	T3	M0
TCGA-G3-	520	0	58	MALE	G3	Stage II	T2	M0
TCGA-ES-	688	1	80	MALE	G2	Stage I	T1	MX
TCGA-FV-	729	0	80	MALE	G2	Stage I	T1	MX
TCGA-DD	1567	0	59	MALE	G3	Stage I	T1	M0
TCGA-RC-	468	0	42	MALE	G3	Stage II	T2	M0
TCGA-ZP-	743	0	51	MALE	G2	unknow	T2	MX
TCGA-DD	1562	0	69	FEMALE	G2	Stage I	T1	M0
TCGA-DD	1145	0	70	MALE	G3	Stage IIIA	T3a	M0
TCGA-2Y-	3675	0	49	MALE	G1	Stage IIIA	T3	M0
TCGA-G3-	65	1	53	FEMALE	G3	Stage IIIA	T3a	M0
TCGA-DD	693	0	68	FEMALE	G1	Stage II	T2	MX
TCGA-FV-	581	1	75	MALE	G1	Stage I	T1	M0
TCGA-DD	1049	0	68	FEMALE	G3	Stage II	T2	M0
TCGA-DD	660	1	75	FEMALE	G3	Stage IIIA	T3a	M0
TCGA-CC-	299	1	47	MALE	G2	Stage II	T2	M0
TCGA-DD	2017	0	72	FEMALE	G2	Stage I	T1	M0
TCGA-DD	3437	0	77	FEMALE	G2	Stage II	T2	M0
TCGA-5R-	46	1	65	FEMALE	G2	Stage II	T2	M0
TCGA-DD	2728	0	38	MALE	G2	Stage I	T1	M0
TCGA-G3-	1636	0	63	FEMALE	G3	Stage I	T1	M0
TCGA-G3-	430	0	31	MALE	G1	Stage I	T1	MX
TCGA-DD	3125	1	66	FEMALE	G2	Stage III	T3	M0
TCGA-DD	171	1	63	FEMALE	G4	Stage I	T1	M0
TCGA-BC-	421	0	75	FEMALE	G3	Stage II	T2	M0
TCGA-ED-	427	0	29	MALE	G2	Stage IIIA	T3a	M0
TCGA-FV-	1	0	51	FEMALE	G2	Stage II	T2	M0
TCGA-DD	1495	0	23	MALE	G3	Stage III	T3	M0
TCGA-FV-	1852	1	70	FEMALE	unknow	Stage II	T2	M0
TCGA-MI-	291	0	64	MALE	G3	Stage I	T1	M0
TCGA-DD	3104	0	32	MALE	G3	Stage I	T1	M0
TCGA-DD	115	1	64	FEMALE	G4	Stage I	T1	M0
TCGA-DD	575	0	65	MALE	G3	Stage I	T1	M0
TCGA-EP-	363	0	73	MALE	G2	Stage IIIA	T3a	MX
TCGA-BC-	1490	1	74	FEMALE	G3	Stage IIIA	T3	M0
TCGA-FV-	848	0	76	FEMALE	G2	Stage II	T2	M0
TCGA-UB-	601	0	24	MALE	G3	Stage II	T2	MX
TCGA-ZP-	706	0	72	MALE	G1	unknow	T1	MX
TCGA-MI-	747	0	77	MALE	unknow	unknow	unknow	MX
TCGA-G3-	698	0	59	MALE	G2	Stage I	T1	M0
TCGA-DD	137	0	73	FEMALE	G2	Stage I	T1	M0
TCGA-ED-	408	0	60	FEMALE	G2	Stage IIIA	T3a	M0
TCGA-BC-	547	1	69	MALE	G3	Stage IIIA	T3a	M0
TCGA-DD	1531	0	72	MALE	G2	Stage I	T1	M0
TCGA-2Y-	1624	1	51	MALE	G2	Stage I	T1	MX

TCGA-XR-	1030	0	49	FEMALE	G3	Stage IIIB	T3	M0
TCGA-2Y-	1452	0	68	MALE	G2	Stage I	T1	MX
TCGA-DD	381	1	48	MALE	G4	Stage I	T1	M0
TCGA-BC-	153	1	80	MALE	G2	unknow	T3	MX
TCGA-2Y-	2532	1	54	FEMALE	G1	Stage I	T1	MX
TCGA-DD	1066	0	70	FEMALE	G3	Stage I	T1	M0
TCGA-CC-	87	1	61	FEMALE	G1	Stage IIIC	T4	M0
TCGA-LG-	387	0	79	FEMALE	G2	Stage II	T2	M0
TCGA-G3-	476	0	58	MALE	G2	Stage I	T1	M0
TCGA-DD	432	1	50	MALE	G3	Stage II	T2	M0
TCGA-KR-	407	0	61	FEMALE	G1	Stage II	T2	M0
TCGA-DD	760	0	54	FEMALE	G2	Stage II	T2	M0
TCGA-DD	2455	0	56	MALE	G3	Stage II	T2	M0
TCGA-DD	16	1	72	MALE	G3	Stage I	T1	M0
TCGA-G3-	935	0	79	FEMALE	G2	Stage IIIB	T3b	M0
TCGA-WJ-	345	0	68	FEMALE	G2	Stage I	T1	MX
TCGA-CC-	315	1	36	MALE	G1	Stage IIIA	T3	M0
TCGA-G3-	361	0	65	MALE	G1	Stage I	T1	MX
TCGA-DD	425	1	53	MALE	G3	Stage I	T1	M0
TCGA-DD	1685	1	61	MALE	G2	Stage I	T1	M0
TCGA-G3-	1553	0	45	FEMALE	G2	Stage IIIA	T3	M0
TCGA-G3-	780	0	53	MALE	G2	Stage IIIA	T3a	M0
TCGA-HP-	752	1	90	FEMALE	unknow	unknow	TX	M0
TCGA-DD	419	1	67	MALE	G3	Stage IIIB	T3b	M0
TCGA-BC-	352	0	73	MALE	G3	Stage IIIA	T3	MX
TCGA-2Y-	36	1	70	MALE	G2	Stage II	T2	MX
TCGA-WX	615	0	64	FEMALE	G3	Stage I	T1	MX
TCGA-DD	601	1	43	FEMALE	G2	Stage II	T2	M0
TCGA-DD	1345	0	54	MALE	G3	Stage I	T1	M0
TCGA-2Y-	1168	0	81	FEMALE	G2	Stage I	T1	MX
TCGA-DD	1005	1	71	FEMALE	G2	Stage IIIC	T1	M0
TCGA-DD	802	1	77	MALE	G2	Stage IIIA	T3a	M0
TCGA-CC-	211	0	55	MALE	G2	Stage II	T2	M0
TCGA-DD	2245	0	65	MALE	G1	Stage I	T1	M0
TCGA-DD	1970	0	56	MALE	G2	Stage IIIA	T3	M0
TCGA-G3-	452	1	52	FEMALE	G3	Stage I	T1	M0
TCGA-BC-	8	0	66	FEMALE	G2	Stage I	T1	M0
TCGA-DD	223	1	40	MALE	G3	Stage IV	T3a	M1
TCGA-DD	2513	0	44	MALE	G2	Stage I	T1	M0
TCGA-UB-	535	0	62	FEMALE	G2	Stage II	T2b	M0
TCGA-ZS-	341	0	55	MALE	G2	Stage II	T2	MX
TCGA-ES-	438	1	54	MALE	G2	Stage I	T1	MX
TCGA-G3-	56	1	70	MALE	G2	Stage IIIB	T3b	MX
TCGA-DD	1085	0	43	FEMALE	G3	Stage I	T1	M0
TCGA-CC-	262	1	59	MALE	G3	Stage IIIA	T3	M0
TCGA-DD	587	0	48	MALE	G3	Stage I	T1	M0
TCGA-FV-	12	0	52	MALE	G2	Stage I	T1	M0
TCGA-DD	12	1	58	MALE	G3	Stage II	T2	M0
TCGA-DD	2015	0	54	MALE	G2	Stage I	T1	M0
TCGA-K7-	359	0	61	MALE	G2	Stage II	T2a	MX
TCGA-ED-	406	0	59	FEMALE	G3	Stage IIIA	T3a	M0
TCGA-DD	3478	0	73	FEMALE	G3	Stage I	T1	M0
TCGA-DD	14	1	73	MALE	G2	Stage I	T1	M0
TCGA-WX	756	0	61	MALE	G1	Stage II	T2	MX
TCGA-ED-	406	0	35	MALE	G3	Stage IIIA	T3a	M0
TCGA-BC-	770	1	52	FEMALE	G2	Stage IIIA	T3a	MX
TCGA-DD	474	0	63	MALE	G2	Stage I	T1	M0
TCGA-MR	229	0	58	MALE	G1	Stage I	T1	MX

TCGA-LG-	366	0	68	MALE	G2	Stage IIIA	T3a	M0
TCGA-FV-	10	0	84	FEMALE	G2	Stage I	T1	M0
TCGA-5C-	328	0	58	MALE	G2	Stage II	T2	M0
TCGA-BC-	308	1	66	FEMALE	G2	unknow	T3	MX
TCGA-DD	410	1	81	FEMALE	G4	Stage I	T1	MX
TCGA-DD	728	0	71	MALE	G2	Stage I	T1	M0
TCGA-BW	0	0	63	MALE	G3	Stage I	T1	MX
TCGA-DD	391	0	25	MALE	G2	Stage I	T1	M0
TCGA-DD	453	0	55	MALE	G3	Stage I	T1	M0
TCGA-FV-	366	1	38	FEMALE	G2	Stage I	T1	MX
TCGA-RC-	588	0	53	MALE	G2	Stage II	T2	M0
TCGA-DD	658	0	66	MALE	G2	Stage I	T1	M0
TCGA-DD	1372	1	76	MALE	G3	Stage I	T1	M0
TCGA-G3-	180	0	71	MALE	G2	Stage I	T1	M0
TCGA-DD	564	0	51	FEMALE	G4	Stage I	T1	M0
TCGA-DD	662	0	69	MALE	G3	Stage I	T1	M0
TCGA-WX	556	1	33	FEMALE	G2	Stage IIIA	T3a	MX
TCGA-NI-	1791	1	71	MALE	G1	Stage IIIA	T3	MX
TCGA-2Y-	1516	0	45	MALE	G1	Stage II	T2	MX
TCGA-BC-	1351	0	62	FEMALE	G2	Stage IIIA	T3	M0
TCGA-2Y-	555	1	59	FEMALE	G3	Stage I	T1	MX
TCGA-EP-	330	0	62	MALE	G1	Stage I	T1	MX
TCGA-DD	415	0	64	MALE	G3	Stage I	T1	M0
TCGA-DD	2131	1	76	FEMALE	G1	Stage I	T1	M0
TCGA-DD	785	1	70	MALE	G2	Stage I	T1	M0
TCGA-DD	2232	0	56	FEMALE	G3	Stage I	T1	M0
TCGA-G3-	354	0	67	MALE	G2	Stage II	T2	M0
TCGA-DD	478	0	60	MALE	G2	Stage I	T1	M0
TCGA-2Y-	633	1	85	FEMALE	G2	unknow	T1	MX
TCGA-G3-	416	1	64	MALE	G2	Stage I	T1	M0
TCGA-RC-	15	0	20	FEMALE	G2	Stage IVA	T1	M0
TCGA-DD	608	0	49	FEMALE	G1	Stage I	T1	M0
TCGA-DD	195	1	69	MALE	G3	Stage II	T2	M0
TCGA-DD	1219	0	73	FEMALE	G2	Stage I	T1	M0
TCGA-CC-	365	0	58	MALE	G1	Stage IIIA	T3	M0
TCGA-DD	722	0	69	MALE	G3	Stage I	T1	M0
TCGA-DD	1233	0	66	FEMALE	G3	Stage I	T1	M0
TCGA-DD	1694	1	78	FEMALE	G2	Stage I	T1	M0
TCGA-DD	1202	0	50	MALE	G4	Stage I	T1	M0
TCGA-K7-	519	0	66	MALE	G1	Stage I	T1	MX
TCGA-DD	2317	0	35	MALE	G2	Stage I	T1	M0
TCGA-G3-	768	1	44	MALE	G2	Stage II	T2	M0
TCGA-MR	330	0	34	MALE	G3	Stage I	T1	MX
TCGA-DD	552	0	52	MALE	G3	Stage I	T1	M0
TCGA-DD	899	1	56	FEMALE	G3	Stage I	T1	M0
TCGA-BW	0	0	26	FEMALE	G3	Stage IV	T2	M1
TCGA-CC-	279	1	51	FEMALE	G2	Stage II	T2	M0
TCGA-G3-	585	0	61	MALE	G2	Stage I	T1	M0
TCGA-EP-	237	0	70	MALE	G2	Stage I	T1	MX
TCGA-2Y-	2442	0	68	MALE	G2	Stage I	T1	MX
TCGA-RC-	640	0	47	FEMALE	G3	Stage I	T1	M0
TCGA-YA-	412	1	68	MALE	G3	Stage IIIA	T3a	MX
TCGA-DD	535	1	45	MALE	G2	Stage I	T1	M0
TCGA-O8-	538	0	54	MALE	G2	Stage I	T1	MX
TCGA-ZP-	717	0	67	FEMALE	G1	unknow	T1	MX
TCGA-DD	349	1	73	MALE	G3	Stage IIIA	T3	M0
TCGA-DD	3258	1	72	FEMALE	G2	Stage II	T2	M0
TCGA-RC-	22	0	74	FEMALE	G2	Stage IIIA	T3	MX

TCGA-DD	672	0	66	MALE	G3	Stage IIIA	T3a	M0
TCGA-K7-	631	0	64	MALE	G1	Stage I	T1	MX
TCGA-CC-	300	1	18	MALE	G1	Stage IIIC	T3	M0
TCGA-G3-	744	0	58	MALE	G1	Stage I	T1	M0
TCGA-DD	373	1	60	MALE	G3	Stage II	T2	M0
TCGA-DD	2301	0	68	MALE	G1	Stage I	T1	M0
TCGA-G3-	412	0	58	FEMALE	G2	Stage II	T2	M0
TCGA-ZP-	1088	1	59	MALE	G1	unknow	T1	MX
TCGA-DD	1531	0	53	MALE	G3	Stage I	T1	M0
TCGA-DD	469	1	52	MALE	G4	Stage II	T2	M0
TCGA-CC-	303	1	61	MALE	G2	Stage IIIA	T3	M0
TCGA-2V-A95S		0	unknow	MALE	G3	Stage II	T2	MX
TCGA-2Y-	1229	1	58	MALE	G2	Stage I	T1	MX
TCGA-DD	428	0	72	MALE	G2	Stage I	T1	M0
TCGA-DD	233	1	65	MALE	G2	Stage IIIA	T3b	M0
TCGA-KR-	657	0	64	MALE	G1	Stage I	T1	M0
TCGA-DD	347	0	51	MALE	G2	Stage I	T1	M0
TCGA-DD	2102	0	75	MALE	G2	Stage II	T2	M0
TCGA-DD	1302	0	32	MALE	G3	Stage I	T1	M0
TCGA-GJ-	31	1	75	FEMALE	G2	Stage II	T2	MX
TCGA-DD	664	0	45	MALE	G3	Stage I	T1	M0
TCGA-DD	574	0	50	MALE	G3	Stage I	T1	M0
TCGA-FV-	247	1	81	FEMALE	G2	Stage II	T2	MX
TCGA-BC-	1135	1	72	FEMALE	unknow	unknow	T2	MX
TCGA-2Y-	724	1	58	MALE	G2	unknow	T2	MX
TCGA-ED-	6	0	48	FEMALE	G3	Stage II	T2	M0
TCGA-DD	11	1	75	MALE	G2	Stage II	T2	M0
TCGA-G3-	632	0	60	FEMALE	G3	Stage I	T1	MX
TCGA-KR-	906	0	57	MALE	G1	Stage I	T1	M0
TCGA-5R-	337	0	17	FEMALE	G3	Stage IIIA	T3a	M0

N	vascular_tu	child_pugh	eastern_cancer_oncology_group
N0	Micro	A	0
N0	None	unknow	unknow
N0	None	unknow	0
N0	unknow	unknow	2
N0	Micro	A	0
N0	None	A	0
N0	None	A	0
N0	unknow	unknow	2
N0	None	B	0
N0	None	A	0
N0	None	A	2
N0	None	A	0
NX	Micro	unknow	unknow
N0	Micro	A	2
N0	Micro	A	0
N0	unknow	A	0
N0	None	A	1
NX	unknow	unknow	0
N0	None	B	0
N0	Micro	A	0
N0	Micro	A	1
N0	None	A	0
N0	Micro	unknow	unknow
NX	unknow	unknow	unknow
NX	None	A	1
NX	unknow	unknow	2
N0	None	A	0
NX	None	A	1
N0	None	unknow	unknow
N0	None	A	0
N0	Micro	A	0
N0	None	unknow	1
N0	None	unknow	unknow
N0	Micro	A	0
N0	unknow	unknow	3
NX	Micro	A	1
N0	None	A	0
N0	unknow	unknow	unknow
N0	unknow	unknow	unknow
NX	Micro	unknow	unknow
NX	None	B	0
N0	Micro	unknow	unknow
NX	None	A	0
N0	unknow	unknow	2
N0	None	A	0
N0	None	unknow	0
N0	None	unknow	unknow
NX	None	A	2
N0	Micro	unknow	unknow
N0	None	A	1
N0	None	A	1
N0	None	A	0
NX	None	unknow	unknow
N0	None	A	0
N0	None	A	0
N0	unknow	unknow	3
NX	None	unknow	1

NX	None	unknow	1
N0	unknow	unknow	1
N0	None	B	2
NX	None	unknow	unknow
N0	Micro	A	1
N0	Micro	A	0
N0	unknow	unknow	unknow
N0	None	A	0
NX	unknow	unknow	0
N0	None	B	1
NX	Micro	unknow	unknow
N0	unknow	unknow	unknow
N0	None	A	1
N0	None	A	0
N0	None	A	1
N0	None	A	1
NX	None	unknow	0
NX	None	A	0
N0	unknow	unknow	3
N0	None	unknow	0
N0	None	A	0
N0	unknow	unknow	4
NX	None	A	0
NX	None	A	0
N0	unknow	unknow	2
N0	None	unknow	unknow
N0	None	A	0
NX	None	unknow	unknow
N0	None	A	0
N0	None	A	1
N0	Micro	A	0
NX	None	A	0
N0	unknow	A	unknow
NX	unknow	A	1
N0	Micro	A	0
NX	None	A	0
NX	Micro	A	0
N0	None	A	1
NX	None	unknow	unknow
N0	None	A	0
N0	unknow	unknow	2
N0	None	A	0
N0	Macro	A	0
NX	Micro	A	1
N0	Micro	A	1
N0	Micro	unknow	unknow
N0	Micro	A	0
NX	Micro	unknow	unknow
N0	unknow	unknow	3
N0	None	A	0
N0	None	A	0
NX	unknow	unknow	unknow
N0	unknow	unknow	3
N0	Micro	A	0
N0	None	unknow	3
NX	Micro	A	unknow
N0	None	A	1
NX	None	unknow	unknow

N0	None	A	0
NX	None	unknow	unknow
NX	Micro	unknow	unknow
N0	None	A	0
N0	Macro	unknow	unknow
N0	Micro	A	0
N0	None	unknow	unknow
N0	Micro	A	2
N0	None	A	0
N0	None	unknow	0
NX	unknow	A	unknow
N0	None	A	1
N0	None	A	0
N0	Micro	unknow	unknow
N0	Micro	A	0
N0	None	unknow	0
NX	Micro	unknow	unknow
N0	None	A	1
N0	unknow	A	1
N0	None	A	0
unknow	Micro	A	1
NX	Micro	unknow	0
NX	None	B	unknow
N0	Micro	B	1
N0	unknow	unknow	unknow
N0	None	A	0
NX	None	A	1
NX	None	A	0
NX	Micro	A	0
N0	None	A	1
NX	None	A	0
N0	None	unknow	unknow
NX	Micro	A	1
N1	unknow	unknow	0
N0	Micro	A	1
N0	None	A	0
NX	Micro	unknow	0
N0	Micro	unknow	unknow
NX	None	A	1
NX	None	A	0
N0	unknow	unknow	unknow
NX	Micro	A	unknow
N0	Micro	B	1
N0	unknow	unknow	3
N0	None	A	0
N0	None	A	0
N0	None	A	1
N0	None	A	0
N0	None	A	1
N0	Micro	unknow	unknow
N0	None	unknow	0
NX	Micro	A	1
N0	Micro	A	0
NX	Macro	unknow	0
NX	unknow	unknow	unknow
N0	None	A	0
N0	None	A	0
N0	Micro	unknow	unknow

N0	None	A		0
N0	Micro	unknow	unknow	
N0	None	A		0
NX	unknow	unknow	unknow	
NX	None	A		0
NX	unknow	unknow	unknow	
NX	None	A		0
N0	None	A		0
N0	None	A		0
N0	None	B		2
NX	None	unknow		0
N0	None	unknow		3
N0	None	unknow		0
N0	unknow	unknow		4
NX	None	A		1
NX	None	A		1
N0	None	unknow	unknow	
N0	None	A		0
N0	Micro	A		1
NX	Micro	A		0
N0	None	A		0
N0	None	A		0
N0	unknow	unknow		0
N0	Micro	A		0
NX	Micro	A		0
N0	unknow	unknow	unknow	
N0	Micro	A		0
N0	None	A		0
N0	unknow	unknow		2
N0	None	B		1
N0	None	A		1
N0	Micro	A		1
N0	None	A		1
N0	None	A		0
NX	None	A		0
N0	None	A		1
N0	Macro	A		1
NX	Micro	unknow	unknow	
N0	Micro	unknow	unknow	
NX	None	unknow	unknow	
N0	Macro	B		1
N0	unknow	unknow	unknow	
N0	None	A		1
N0	None	B		0
N0	None	A		1
N0	None	A		0
NX	unknow	A		0
N0	Macro	unknow	unknow	
NX	Micro	unknow	unknow	
NX	Micro	A		0
NX	None	A		0
NX	Micro	B		1
NX	None	B		0
N0	None	A		0
N0	Micro	unknow	unknow	
NX	Micro	A	unknow	
N0	None	A		0
NX	None	C		0

N0	None	A	1
N0	None	A	0
N0	Micro	A	1
NX	None	unknow	unknow
NX	unknow	unknow	2
N0	None	A	0
N0	None	unknow	unknow
NX	Micro	A	0
N0	None	A	0
N0	None	A	0
N0	Micro	unknow	unknow
N0	None	A	0
N0	Micro	A	1
N0	Micro	A	0
N0	Macro	A	0
NX	None	A	1
N0	unknow	unknow	3
NX	None	A	0
N0	Macro	A	0
N0	None	A	0
N0	None	A	0
N0	None	A	0
NX	unknow	unknow	0
N0	Micro	unknow	1
N0	Macro	unknow	0
NX	unknow	A	0
NX	None	A	1
NX	None	B	2
N0	None	A	0
N0	None	A	2
N1	None	B	2
NX	Micro	A	1
N0	unknow	unknow	2
N0	None	A	0
N0	Micro	A	0
N0	None	A	0
NX	None	unknow	unknow
N0	Micro	A	1
N0	None	A	1
N0	Micro	A	0
NX	Micro	A	0
NX	None	A	1
NX	Macro	A	0
N0	None	A	0
N0	unknow	unknow	2
N0	None	A	1
NX	None	unknow	unknow
N0	None	A	0
N0	None	A	0
NX	Micro	A	0
N0	Micro	unknow	unknow
N0	None	A	0
N0	None	A	1
NX	Macro	A	0
N0	Micro	unknow	unknow
N0	Micro	unknow	unknow
N0	None	A	0
NX	unknow	unknow	unknow

N0	None	unknow	unknow
NX	None	unknow	unknow
N0	Micro	A	0
NX	Micro	unknow	unknow
N0	None	A	1
N0	None	A	1
NX	None	unknow	unknow
N0	None	unknow	1
N0	Micro	A	0
NX	None	unknow	1
N0	None	B	0
N0	Micro	A	0
N0	None	A	0
N0	None	A	1
N0	None	A	1
N0	None	A	1
NX	Macro	A	2
NX	None	unknow	0
NX	Micro	unknow	2
NX	None	unknow	unknow
N0	None	unknow	unknow
NX	None	B	unknow
N0	None	A	0
N0	None	A	0
NX	None	A	1
N0	None	A	1
N0	Macro	A	1
N0	None	A	1
NX	unknow	unknow	2
N0	Micro	A	1
N1	None	A	unknow
N0	None	A	0
N0	Micro	A	0
N0	None	A	0
N0	unknow	unknow	3
N0	None	A	0
N0	Macro	A	0
NX	None	A	1
N0	Micro	A	0
NX	None	unknow	unknow
N0	None	A	0
N0	None	A	1
N0	None	unknow	unknow
N0	None	A	0
N0	None	A	0
N0	Micro	unknow	2
N0	unknow	unknow	2
N0	None	A	0
N0	None	unknow	1
NX	unknow	unknow	1
N0	None	A	0
N0	None	unknow	unknow
N0	Macro	B	1
NX	None	A	unknow
NX	None	A	0
N0	None	B	2
N0	Micro	A	0
NX	None	A	1

N0	None	A		1
NX	unknow	unknow	unknow	
N1	unknow	unknow		3
NX	unknow	A		0
N0	Macro	A		0
N0	None	A		1
N0	None	A		1
NX	None	A		1
N0	Micro	A		0
N0	Micro	A		0
N0	unknow	unknow		3
NX	Micro	A		4
NX	unknow	unknow		1
N0	None	A		0
N0	Macro	A		0
N0	None	unknow	unknow	
N0	None	A		0
N0	Micro	A		2
N0	None	A		0
NX	Micro	unknow	unknow	
N0	None	A		0
N0	None	A		0
N0	Micro	unknow	unknow	
NX	None	unknow	unknow	
NX	unknow	unknow	unknow	
NX	Micro	unknow	unknow	
N0	None	A		1
NX	None	A		0
N0	None	unknow	unknow	
N0	Micro	B		1
