# Supplementary figures and images for: Construction of a novel immune-related lncRNA signature and its potential to predict the immune status of patients with hepatocellular carcinoma
Source: BMC Cancer. 2021 Dec 19;21:1347. doi: 10.1186/s12885-021-09059-x (PMC8684648; doi:10.1186/s12885-021-09059-x)

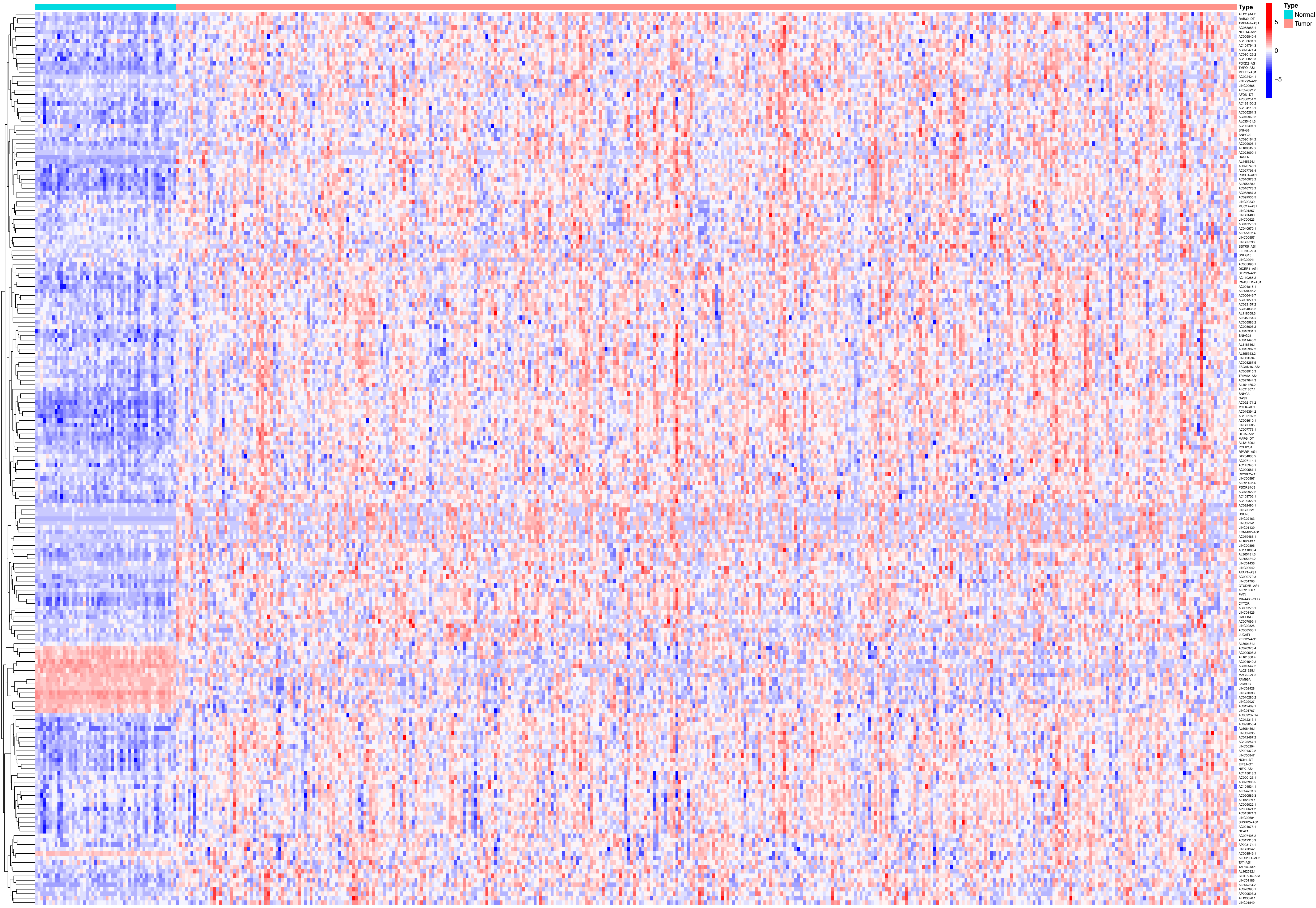

Supplement: Supplementary file 4 — Additional file 4. [file 12885_2021_9059_MOESM4_ESM.pdf]
